# Clinically accurate diagnosis of Alzheimer’s disease via multiplexed sensing of core biomarkers in human plasma

**DOI:** 10.1038/s41467-019-13901-z

**Published:** 2020-01-08

**Authors:** Kayoung Kim, Min-Ji Kim, Da Won Kim, Su Yeong Kim, Steve Park, Chan Beum Park

**Affiliations:** Department of Materials Science and Engineering, Korea Advanced Institute of Science and Technology (KAIST), 335 Science Road, Daejeon, 305-701 Republic of Korea

**Keywords:** Biosensors, Alzheimer's disease, Carbon nanotubes and fullerenes

## Abstract

Alzheimer’s disease (AD) is the most prevalent neurodegenerative disorder, affecting one in ten people aged over 65 years. Despite the severity of the disease, early diagnosis of AD is still challenging due to the low accuracy or high cost of neuropsychological tests and neuroimaging. Here we report clinically accurate and ultrasensitive detection of multiple AD core biomarkers (t-tau, p-tau_181_, Aβ_42_, and Aβ_40_) in human plasma using densely aligned carbon nanotubes (CNTs). The closely packed and unidirectionally aligned CNT sensor array exhibits high precision, sensitivity, and accuracy, evidenced by a low coefficient of variation (<6%), a femtomolar-level limit of detection, and a high degree of recovery (>93.0%). By measuring the levels of t-tau/Aβ_42_, p-tau_181_/Aβ_42_, and Aβ_42_/Aβ_40_ in clinical blood samples, the sensor array successfully discriminates the clinically diagnosed AD patients from healthy controls with an average sensitivity of 90.0%, a selectivity of 90.0%, and an average accuracy of 88.6%.

## Abstract (web summary)

Detection of Alzheimer’s disease (AD) biomarkers from patients’ blood is challenging because these are present in very low concentrations in the plasma. Here the authors develop a sensor array of densely aligned single-walled carbon nanotubes for clinically accurate detection of femtomolar AD biomarkers in human plasma samples.

## Introduction

Alzheimer’s disease (AD) is the world’s most common neurodegenerative disorder, causing severe cognitive decline and irreversible memory loss^1^. AD affects more than 10% of the population over the age of 65 years worldwide and the incidence of AD will increase by more than threefold within the next 50 years^2^. Currently, clinical diagnosis of AD relies largely on neuropsychological tests and neuroimaging, but the low accuracy of cognitive assessments and the high cost of brain imaging leave a large number of AD patients diagnosed late or not at all^3^. AD diagnosis at a preclinical stage is critical, because treatment after the onset of clinical symptoms cannot stop or reverse the disease progression. Early diagnosis can reduce the risk of suffering AD by one-third, according to recent reports^4^.

The major pathological hallmarks of AD are the formation of neuritic plaques composed of amyloid-β (Aβ) peptides and neurofibrillary tangles^5,6^. Aβ peptide aggregation initiates the pathogenic cascade—including hyper-phosphorylation of microtubule-associated tau protein and the formation of intracellular tau aggregates—which leads to synaptic dysfunction, neuronal death, and loss of cognitive capacity^7^. According to longitudinal and cross-sectional studies^8,9^, the level of Aβ_42_, phosphorylated tau (p-tau) proteins, and total tau (t-tau) proteins start to change almost 10–15 years before the appearance of AD symptoms. Clinical studies have reported that the concentration of t-tau protein in AD patients’ cerebrospinal fluid (CSF) is two to three times higher than that of normal controls, whereas Aβ_42_ decreases by ~40% in AD patients when compared with those of healthy individuals^10^. Recently, a strong correlation between the levels of AD biomarkers in blood plasma and pathological changes in the brain have been reported^11,12^. As the CSF sampling process via lumbar puncture has serious drawbacks, including invasiveness and lack of accessibility^13^, well-validated blood-based biomarker panels may facilitate minimally invasive assessment of AD patients in primary care settings and continuous monitoring of disease progression. However, the concentrations of biomarkers in plasma are 10- to 10^2^-fold lower than in CSF and blood samples contain high levels of miscellaneous interfering agents, rendering the accurate and reliable detection of blood-based biomarkers difficult^14^.

**Fig. 1:**
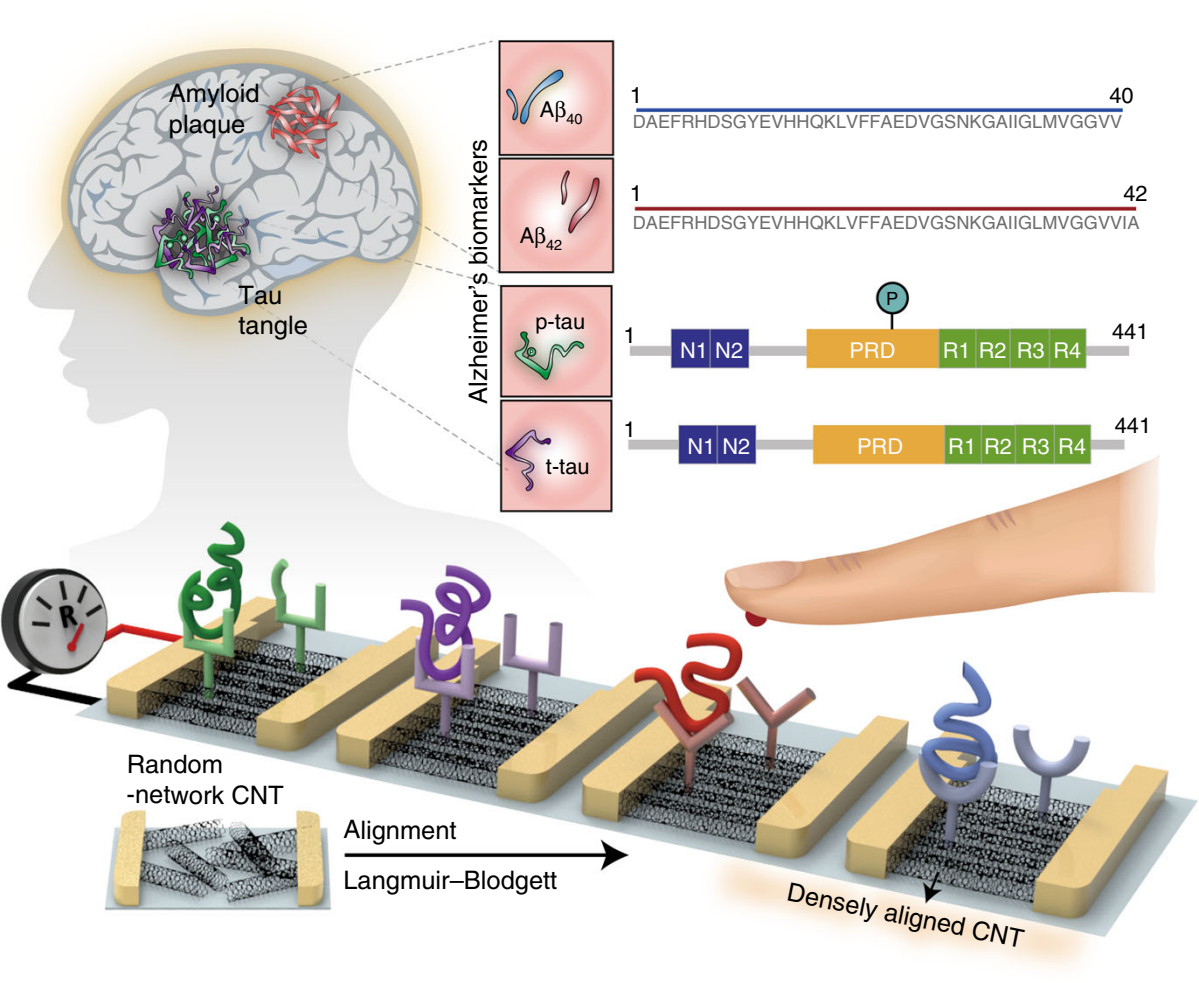
Schematic illustration of a densely aligned CNT sensor array for AD biomarkers. The unidirectional aligned and highly dense CNTs facilitate femtomolar sensitivity and high sensor-to-sensor reliability. The densely aligned CNT sensor array exhibited diagnostic accuracy of ~88.6% in discriminating AD patients from healthy individuals.

Here we report on a multiplexed electrical sensing platform for clinically accurate detection of AD core biomarkers in human plasma, as depicted in Fig. 1. We have designed the sensor array by employing a densely aligned single-walled carbon nanotube (CNT) thin film as a transducer. The highly dense and unidirectionally aligned CNT sensor array can reliably detect target analytes down to femtomolar concentrations because of its low density of tube-to-tube junctions and uniform number of CNTs per device. We achieve the lowest-ever limit of detection (LOD) toward AD biomarkers among electrical sensing platforms reported so far^15–17^. The sensor array can accurately measure the concentrations of AD biomarkers in human plasma and selectively recognize multiple biomarkers without any cross-reactivity. Furthermore, we substantiate that the densely aligned CNT sensor array can discriminate clinically diagnosed AD patients from normal controls by estimating the levels of composite AD biomarkers (t-tau/Aβ_42_, p-tau/Aβ_42_, and Aβ_42_/Aβ_40_) in human plasma.

## Results

### Characterization of densely aligned CNT sensor array

**Fig. 2:**
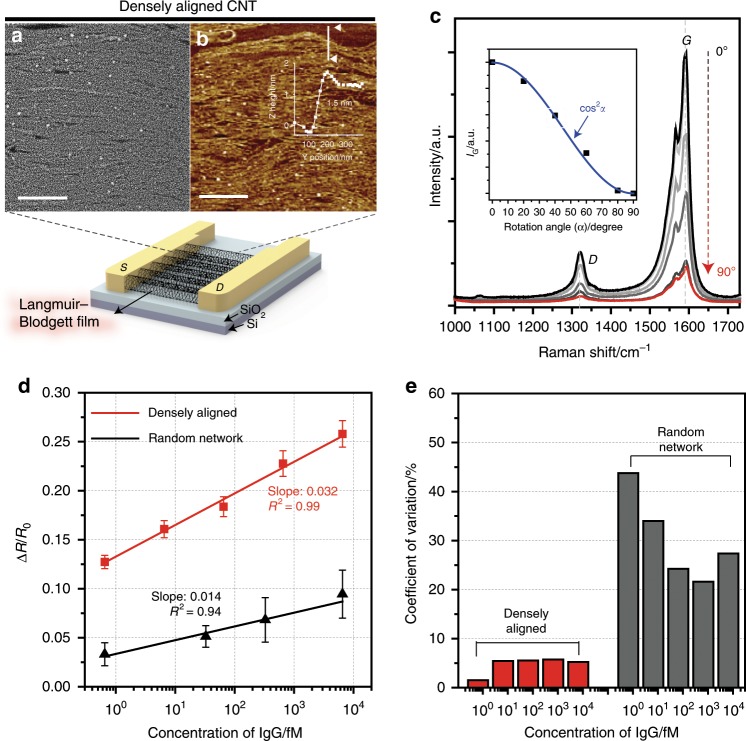
Characterization of densely aligned CNT sensor array. **a** SEM and **b** AFM images of densely aligned CNT film. The scale bars in SEM and AFM images indicate 250 nm and 500 nm, respectively. **c** Polarized Raman spectra of the densely aligned CNT film recorded at various angles between 633 nm incident laser and an alignment direction of CNT film. The inset shows the angular dependence of the Raman intensity at 1595 cm^−1^. The blue solid line fits cos^2^α function. **d** Sensing performance of densely aligned CNT device in comparison with random-network CNT devices that have similar CNT densities. The densities of densely aligned and random-network CNT samples were 345 and 339 CNTs μm^−1^, respectively. For each data point, a different set of devices were used. Data reproducibility was confirmed by two additional experiments. All reported values represent the mean ± SD. **e** The values of coefficient of variation (CV) of resistance change in the densely aligned and random-network CNT device array. The CV of densely aligned device array was approximately six times lower than that in random-network CNT device array. Source data are provided as a Source Data file.

We prepared densely aligned CNT films using the Langmuir–Blodgett (LB) transfer method, which involves compressing the CNTs at the water–air interface and their subsequent transfer onto a silicon substrate (Supplementary Fig. 1). To obtain densely packed, well-aligned CNT monolayers, we repeated the compression–retraction of mobile bars applying uniaxial force to the poly [(m-phenylenevinylene)-co-(2,5-dioctoxy-p-phenylenevinylene)] (PmPV)-wrapped CNTs floating on water sub-phase. The detailed underlying mechanism about the formation of densely aligned CNT monolayer film is described in the Supplementary Fig. 2. We found that the critical number of LB cycling for the formation of densely aligned CNT monolayer film was ten compression–retraction cycles with gradual increase of the surface pressure for each cycling to eventually reach 35 mN m^−1^ (Supplementary Fig. 3). According to the surface pressure isotherm curves recorded during ten cycles of compression and expansion (Supplementary Fig. 4A), negligible hysteresis was observed in each cycle. In contrast, the compression of CNTs up to the 35 mN m^−1^ at once caused a large hysteresis in the isotherm curve (Supplementary Fig. 4B). These results imply that the multiple cycling procedures inhibited self-aggregation of CNTs and the formation of loops^18^. As shown in Fig. 2a and Supplementary Fig. 5, the CNT films fabricated by repeated LB cycling exhibited highly packed and well-aligned CNTs, whereas a large number of loops appeared in the CNT films transferred after single LB cycling. According to atomic force microscopic analysis of topographical profiles (Fig. 2b), the height of the densely aligned CNT film was ~1.5 nm, which indicates a monolayer of aligned CNTs. To analyze the alignment of CNT films, we employed polarized Raman spectroscopy that allows determination of materials’ anisotropic properties with a high aspect ratio^19^. As shown in Supplementary Fig. 6, the Raman spectra of the random-network CNT film showed a negligible change with the variation of the polarized angle from 0° (laser parallel to the alignment direction) to 90° (laser perpendicular to the alignment direction). In contrast, the densely aligned CNT films exhibited obvious optical anisotropy; the intensity of the G-band in the Raman spectra gradually decreased with the increasing rotation angle (Fig. 2c). This angular (*α*) dependence of the G-band intensity matched well with a cos^2^*α* function, which suggests that the LB-transferred CNT films were unidirectionally aligned. We subsequently annealed the CNT films to remove PmPV moieties on the CNTs, which was confirmed by a Fourier transform infrared analysis (Supplementary Fig. 7). It is noteworthy that LB-deposited CNTs retained their alignment without curling or rolling after heat treatment under Ar atmosphere (Supplementary Fig. 8)

We designed an array of devices by patterning source and drain electrodes on top of the densely aligned CNT films. The CNT channels were modified by analyte-specific antibodies via a carbodiimide-assisted covalent conjugation method (Supplementary Figs. 9–12; see the experimental section for details). We investigated the variations in the resistance of the densely aligned CNT sensor array upon the addition of immunoglobulin G (IgG); as shown in Fig. 2d and Supplementary Fig. 13, the changes in the sensor array’s resistance showed a linear dependency on the logarithmic concentration of IgG. The densely aligned CNT array exhibited high sensitivity toward IgG; its sensitivity was 2.29 times higher than that of the random-network sample having a similar CNT density. In addition, the densely aligned CNT sensor array showed 1.88-fold higher sensitivity than the random-network CNT sample with comparable initial resistance (Supplementary Fig. 14B,D). It is worth noting that the amount of anti-IgG immobilized on CNTs was similar for all arrays (Supplementary Fig. 14A,C). We ascribe these results to the high density of tube-to-tube junctions in the random-network CNT sample (Supplementary Fig. 15), considering the similarity of CNT lengths in the densely aligned CNTs and the random-network films (Supplementary Figs. 16 and 17). The highly resistive tube-to-tube junction increases the initial resistance of CNT devices and renders the sensor array less sensitive to analytes, as noted in the literature^20–22^. We further examined the effect of CNT’s surface coverage on the sensitivity of the sensor array. As shown in Supplementary Fig. 18A, a positive correlation of sensor array’s sensitivity to the surface coverage of CNT film was observed. We ascribe this result to the large amount of immobilized bioreceptors on CNTs with the increasing surface coverage of CNT film (Supplementary Fig. 18B). In addition to high sensitivity, the densely aligned CNT sensor array exhibited a low device-to-device variation. As displayed in Fig. 2e, the coefficient of variation (CV) of changes in the densely aligned CNT device array’s resistance was under 6%, whereas CV values were at least six times higher in the random-network CNT device array. This result is attributed to the uniformity of the number of CNTs per device in the densely aligned CNT array, which was evidenced by the low CV value for the array’s resistance (Supplementary Fig. 19).

### AD core biomarkers detection by densely aligned CNT array

We employed the densely aligned CNT device array to detect AD core biomarkers (t-tau, p-tau_181_, Aβ_42_, and Aβ_40_) by modifying each CNT channel with the recognition element that specifically captured each biomarker. The detailed information about the antibodies used in our sensor arrays is described in Supplementary Table 1 and Supplementary Fig. 20. Supplementary Fig. 21 shows the changes in the resistance of the densely aligned CNT sensor array upon its exposure to the biomarkers. The specific binding of target biomarkers to the corresponding bioreceptors led to an increase in the resistance of sensor array by over 11%, whereas non-target biomarkers (i.e., human IgG) caused no significant responses. It is noteworthy that the densely aligned CNT sensor arrays could detect both native and denatured biomarkers when the epitope of each antibody is unmasked (or undamaged) (Supplementary Fig. 22). To explore the underlying mechanism of the sensor array’s resistance being increased by the target biomarkers, we analyzed the transfer characteristics of the densely aligned CNT device by applying an additional liquid-ion gate (Supplementary Fig. 23A). As shown in Supplementary Fig. 3B-E, the transfer curve of the densely aligned CNT sensor array exhibited typical p-type semiconductor characteristics. The addition of target AD biomarkers to the sensor arrays reduced the source-to-drain currents at the negative gate voltage, leaving the positive gate voltage region unaffected. These results indicate that target AD biomarkers captured by the corresponding recognition elements acted as scattering centers on CNTs^23^ and eventually decreased the array’s conductance.

**Fig. 3:**
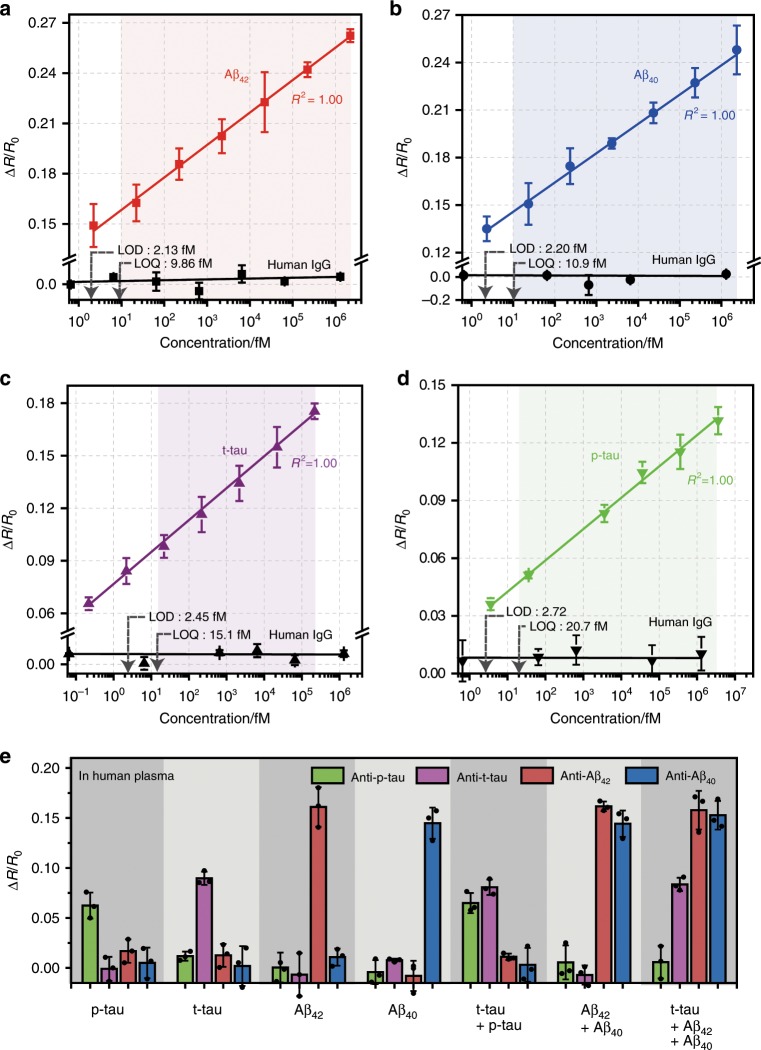
Densely aligned CNT sensor arrays’ sensitivities toward AD core biomarkers. Changes in resistance of the densely aligned CNT sensor array with the increasing concentration of (**a**) Aβ_42_, (**b**) Aβ_40_, (**c**) t-tau, and (**d**) p-tau. Each data point was attained using a different set of devices. The human IgG was used as a negative control. **e** Selectivity of the densely aligned CNT sensor array toward various individual AD biomarkers and their mixtures in human plasma. The concentrations of Aβ_42_ and Aβ_40_ were 22.2 and 23.1 fM, respectively. In the case of t-tau and p-tau, the concentrations were 21.8 fM and 360 fM, respectively. The measurement was performed in triplicate and all reported values represent the mean ± SD. Source data are provided as a Source Data file.

To quantitatively evaluate the sensitivity and accuracy of the densely aligned CNT-based sensor array, we investigated the changes in the sensor array’s resistance by varying the AD biomarkers’ concentrations. We observed a strong linear relationship between the sensor array’s resistance change and the biomarkers’ logarithmic concentration in the range of 10^0^ to 10^6^ fM (Fig. 3a–d). The coefficients of determination (*R*^2^) of the regression line were over 0.99 and the CV values of resistance changes were under 10% (Supplementary Fig. 24). Based on the results of linear regression analysis, the LOD and limit of quantification (LOQ) of the sensor array toward AD biomarkers were estimated at a confidence level of 3.3 and 10, respectively. The values of LOD and LOQ of four AD biomarkers range from 2.13 to 2.72 fM (Supplementary Table 2), which were 10^2^ to 10^3^ times lower than the levels of the biomarkers in AD patients’ blood^11,24,25^. We further examined the accuracy of the sensor array by detecting AD biomarkers spiked in the pooled blood plasma (Supplementary Fig. 25). Linear dependences of the array’s resistance change on the target biomarkers’ logarithmic concentrations were observed (*R*^2^ > 0.99) and the degree of recovery for spiked AD biomarkers was over 93.0%, within the clinically relevant range of 10^1^–10^4^ fM. These results show that the densely aligned sensor array can accurately and reliably detect target AD biomarkers of femtomolar levels without being disturbed by interfering agents in blood plasma.

### Multiplex sensing of AD biomarkers in clinical blood samples

To demonstrate the multiplexed sensing capabilities of the densely aligned CNT sensor array, we prepared a CNT array device modified by different antibodies specific for t-tau, p-tau_181_, Aβ_42_, and Aβ_40_, respectively. Upon the addition of the mixed AD biomarkers, significant changes in resistance were observed only in the CNT devices that contained the targeted AD biomarker-specific recognition elements (Supplementary Fig. 26). In contrast, no obvious response was observed for non-target analytes abundant in human plasma (such as IgG, immunoglobulin M, human albumin serum, and transferrin) despite their nanomolar concentrations. In addition, we investigate the sensor array’s capability to discriminate different Aβ aggregate species (such as monomeric species, oligomers, and fibrillar aggregates). As shown in Supplementary Fig. 27, the densely aligned CNT sensor arrays successfully distinguished between oligomeric and fibrillar Aβ species, respectively, without the interference by other non-target Aβ species. We further performed multiplexed detection of AD biomarkers that were spiked in the pooled blood plasma. As shown in Fig. 3e, the sensor arrays accurately discriminated both single and mixed AD biomarkers down to femtomolar concentrations. Despite the existence of hundreds of non-target proteins in the plasma, the levels of recovery were over 92% in all cases. These findings imply that the densely aligned CNT-based multiplexed sensing platform can selectively recognize AD biomarkers without significant interference from other non-target proteins.

**Fig. 4:**
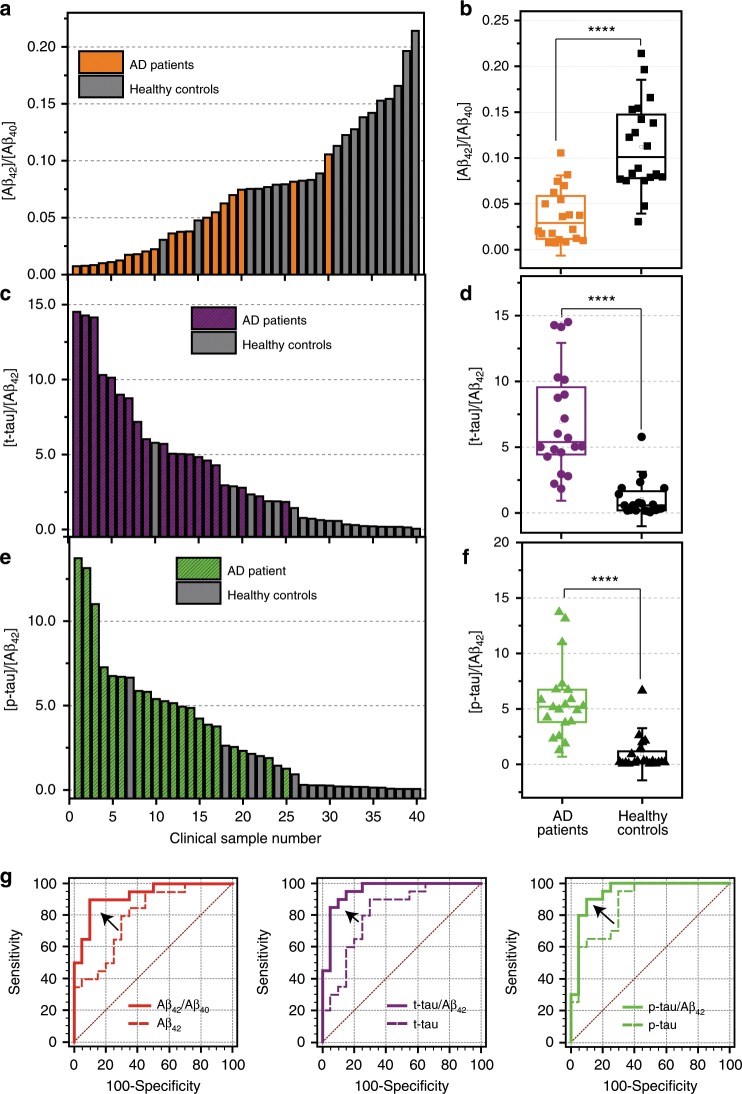
Clinical relevance of the densely aligned CNT sensor array. Waterfall plots and box plots showing the estimated levels of (**a**, **b**) Aβ_42_/Aβ_40_, (**c**, **d**) t-tau/Aβ_42_, and (**e**, **f**) p-tau/Aβ_42_ in the plasma of AD patients (*n* = 20) and healthy controls (*n* = 20). Statistical analysis was carried out by means of one-way analysis of variance (ANOVA). ****p *<* 0.000001. In the boxes, the 25th, 50th (median), and 75th percentiles of the data are indicated. The whiskers represent mean ± 1.5 SD. **g** Receiver operating characteristic curves of the sensor array when composite biomarkers (Aβ_42_/Aβ_40_, t-tau/Aβ_42_, and p-tau/Aβ_42_) and single biomarker (Aβ_42_, t-tau, p-tau) were used as a predictor, respectively. Source data are provided as a Source Data file.

We investigated the clinical applicability of the densely aligned CNT-based sensing platform using samples from AD patients and healthy controls. Detailed demographic characteristics of the subjects are listed in Supplementary Table 3. As shown in Supplementary Fig. 28, we found that the average concentrations of t-tau and p-tau_181_ in plasma samples obtained from AD patients were 2.40 and 3.34 times higher than those in controls, respectively (*p* < 0.001, one-way analysis of variance (ANOVA)). In addition, the estimated average concentration of Aβ_42_ in AD patients’ blood plasma was 2.97 times higher than in normal controls (*p* < 0.001, one-way ANOVA), whereas the level of Aβ_40_ in plasma was similar for both groups (*p* = 0.26, one-way ANOVA). The measured plasma levels of AD biomarkers are listed in Supplementary Table 4, which are comparable to the reported values in previous clinical studies^11,12,24,25^. It is noteworthy that our sensor array could detect both unbound Aβ_42_ and Aβ_42_ bound with other proteins in blood plasma, because the resistance of our sensor array increased upon exposure to the plasma filtrate as well as to the native plasma sample (Supplementary Fig. 29). According to the literature, testing a combination of multiple biomarkers is superior to a single one in predicting individuals at risk of AD among those who are cognitively normal^26^. To this end, we compared the plasma levels of composite biomarkers (t-tau/Aβ_42_, p-tau_181_/Aβ_42_, and Aβ_42_/Aβ_40_) between the AD patients and the control groups. Highly significant differences in the levels of composite biomarkers were found between AD groups and normal controls (*p* < 0.000001, one-way ANOVA), whereas differences in the levels of a single biomarker were only significant at the level of 0.001 (Fig. 4a–f and Supplementary Fig. 28). To evaluate the multiplexed sensor array’s capability to discriminate AD patients from healthy controls, we further conducted receiver operator characteristic (ROC) analyses (Fig. 4g and Supplementary Table 5). The ROC analysis is a standard statistical method to assess diagnostic sensitivity, specificity, and accuracy of the sensing platform (Supplementary Fig. 30)^27–29^. By employing the composite biomarkers as predictors, we have successfully discriminated AD patients and healthy controls with an average sensitivity of 90.0%, a selectivity of 90.0%, and an average accuracy of 88.6%. In contrast, the sensor array exhibited relatively low sensitivity, selectivity, and accuracy (ranging from 70.0% to 88.3%) when the single biomarker alone was used as a classifying marker. We further investigated the area under the ROC curve (AUC) values for each biomarker. The AUC parameter quantifies the diagnostic accuracy of a sensing platform; the value of 1.0 represents the perfect clinical prediction^30,31^. We found that the estimated AUC values for composite biomarkers were over 0.93, whereas individual biomarkers yielded smaller AUC values ranging from 0.80 to 0.87. Overall, these results show that quantification of t-tau/Aβ_42_, p-tau/Aβ_42_, and Aβ_42_/Aβ_40_ levels in clinical samples based on the densely aligned CNT sensor array can distinguish AD patients from healthy controls with an accuracy of ~90.2%.

## Discussion

According to our results, the unidirectional alignment and dense packing of CNTs via the LB transfer process facilitated high device-to-device reliability (CV < 6%) and femtomolar-level LODs for detecting AD biomarkers. The densely aligned CNT sensor array exhibited the lowest LOD when compared with those of previously reported CNT-based biosensors (Supplementary Fig. 31). LB assembly can produce uniform monolayered films on any substrate without sophisticated nanolithographic techniques^18,32^. The superior sensitivity and high degree of precision enabled the sensing platform to accurately quantify the plasma-based biomarker (i.e., recovery for the spiked biomarkers > 93.0%) despite the existence of hundreds of interfering agents in the blood plasma. The densely aligned CNT sensor array was 10–10^3^ times more sensitive than the commercially available sandwich-type, enzyme-linked immunosorbent assay (Supplementary Fig. 32)^33^. Other sensing platforms based on surface enhanced Raman spectroscopy and fluorescence measurements have been developed for detecting AD biomarkers^34,35^; however, their LOD values were 10- to 10^2^-fold higher than those achieved by our sensor platform. Recently, highly sensitive optical analytic platform based on surface plasmon resonance has been reported^36^; they have validated the clinical relevance of the sensing platform toward Aβ42 in blood plasma, whereas diagnostic accuracy of our sensor array was verified by measuring the plasma levels of four different types of AD biomarkers (Supplementary Fig. 33). Furthermore, previously reported electrical sensing platforms recognized only single AD biomarkers (either Aβ_42_ or Aβ_40_) and exhibited ~10 times lower sensitivities than our sensor array^15–17^. In case of analytic techniques based on optical device or mass spectroscopy^11,37^, the requirement of expensive and specialized equipment limits the usability and simplicity of the sensing platforms. In contrast, our sensor array can be readily developed into a small portable device with an integrated sensor and readout circuitry (Supplementary Table 6).

Simultaneous detection of multiple biomarkers is highly desirable because of the heterogeneity of dementia pathology and the complexity of AD pathogenesis. According to the literature^11,38,39^, Aβ_42_/Aβ_40_ and p-tau_181_/Aβ_42_ ratios are more accurate diagnostic biomarkers than single ones in predicting the progression of AD and differentiating AD patients from normal controls because of the inverse correlation between the levels of tau proteins and Aβ_42_. Furthermore, longitudinal studies have demonstrated that t-tau/Aβ_42_ and p-tau_181_/Aβ_42_ ratios show a notable increase 15 years before the onset of clinical symptoms, but the level of Aβ_42_ begins to decrease only around 10 years before the emergence of the expected symptoms^40^. In the present study, we verified the multiplexed sensing capabilities of the densely aligned CNT sensor array, which selectively recognized AD biomarkers on a femtomolar level without cross-reactivity for non-target biomarkers in the plasma. For clinical validation, we tested clinical plasma samples from AD patients as well as normal controls over an age of 65 years. By estimating the plasma levels of composite biomarkers (Aβ_42_/Aβ_40_, t-tau/Aβ_42_, and p-tau_181_/Aβ_42_), the densely aligned CNT sensor array showed superior diagnostic accuracy that was evidenced by an average sensitivity of 90.0% and selectivity of 90.0%. The AUC values for composite biomarkers were above 0.93, which are higher than the threshold for AD patient relevance^31^. Further studies to improve the sensitivity of our sensor array and additional assessments using a large-scale cohort are needed to translate the proposed sensor array into clinical diagnosis.

In summary, we have synthesized closely packed and unidirectionally aligned CNT arrays for extremely sensitive and selective detection of multiple AD core biomarkers in blood plasma. The densely aligned CNT sensor array exhibited 2.29 times higher sensitivity than the random-network CNT device array due to the low density of tube junctions and the uniformity of the number of CNTs per device. The integration of the four types of bioreceptors into the densely aligned CNTs enabled the sensor array to detect AD core biomarkers down to femtomolar concentrations; the values of LOD were 2.13 fM for Aβ_42_, 2.20 fM for Aβ_40_, 2.45 fM for t-tau, and 2.72 fM for p-tau_181_. In addition to its femtomolar sensitivity, the multiplexed sensor array selectively recognized four different types of core AD biomarkers in the blood plasma. By simultaneously detecting composite biomarkers (i.e., t-tau/ Aβ_42_, p-tau/ Aβ_42_, and Aβ_42_/Aβ_40_) in clinical plasma samples, the sensor array successfully distinguished AD patients from healthy controls (*p* < 0.000001, one-way ANOVA) with an average AUC value of 0.94. This work demonstrates the high potential of the aligned CNT-based, multiplexed, and electrical sensing platform for early AD diagnosis.

## Methods

### Chemicals and materials

Single-walled CNT was obtained from OCSiAl, USA. Human Aβ_42_ peptide, human Aβ_40_ peptide, and human tau_441_ protein were purchased from rPeptide Co., USA. Synthetic p-tau_181_ peptide (sequence Q_165_ANATRIPAKTPPAPKT_phospho_PPSSGEPPKS_191_) was provided by Peptron, Inc., Korea. Monoclonal antibodies reactive to p-tau_181_ (clone M7004D06), C-terminus of Aβ_42_ (Clone 12F4), and C-terminus of Aβ_40_ (Clone 11A50-B10) were purchased from BioLegend, Inc. Antibody (Clone Tau-5) that recognizes all phosphorylated and non-phosphorylated isoforms of tau proteins was obtained from EMD Millipore Co., USA. The epitope of Tau-5 lies within amino acids 210–241 of tau proteins. *N*-hydroxysulfosuccinimide (sulfo-NHE) was purchased from Thermo Fisher Scientific, Inc., USA. All other chemicals were purchased from Sigma-Aldrich Chemical Co., USA.

### Preparation of densely aligned CNT film

We suspended 2.5 mg of CNT and 2.5 mg of PmPV into 25 ml of dichloroethane (DCE) and sonicated the suspension for 1 h using horn sonicator at 70% amplitude. After centrifugation at 31,664 × *g* for 1 h, the supernatant was filtered several times through Teflon filter paper (Sigma-Aldrich Chemical Co., USA), followed by re-dispersion in clean DCE. We diluted the final suspension to 1/6 and sonicated the solution for 10 min before use. Afterwards, we filled deionized water into the LB deposition trough (KSV NIMA, KN 2001, Finland) and dropped 800 μL of CNT solution on the surface of the water. After evaporation of the solvent for 10 min, we repeated the compression–expand cycles with a rate of 15 mm min^−1^. Afterwards, the floated CNTs were transferred to SiO_2_ (100 nm)/Si substrate by pulling up the substrate at a rate of 2 mm min^−1^. The random-network CNT film was fabricated using the spin coating method at 1000 r.p.m. The as-prepared CNT film was annealed at 550 °C for 1.5 h to remove PmPV moieties on the CNTs.

### Fabrication of CNT-based sensor

We patterned CNT devices by depositing metallic source and drain contacts (Cr/Au = 5/50 nm) on top of CNT films using an e-beam evaporator (SNTECK Co. Ltd, Korea). The width and the length of each CNT channel were 1.6 mm and 200 μm, respectively. To block unexpected side responses at the contacts, the metal contacts were passivated by subsequent deposition of additional layers (Cr/SiO_2_ = 5/50 nm). It is noteworthy that we did not use additional etching method to disconnect adjacent CNT device on the Si wafer, because the presence of CNTs between adjacent CNT devices did not affect the resistances of individual CNT devices (Supplementary Fig. 34). Polydimethylsiloxane (PDMS) well was mounted on as-fabricated sensor chip for multiplexed detecting of AD biomarkers.

### Functionalization of CNT film

For introducing the carboxyl group into the surface of CNT film, we exposed the ultraviolet (UV)-ozone to as-prepared CNT device array using UV-ozone cleaner (Ahtech LTS Co., Ltd, Korea). After UV-ozone treatment for 7 min, each CNT device was incubated for 30 min at room temperature in 0.1 M 2-(*N*-morpholino)ethanesulfonic acid (MES) buffer (pH 5.0) solution containing 200 mM 1-ethyl-3-(3-dimethylaminopropyl)carbodiimide and 1 M sulfo-NHE. After that, we washed each CNT device with 10 mM phosphate-buffered saline (PBS, pH 7.4) for removing residual materials. We prepared 300 μg mL^−1^ of antibody solutions (i.e., anti- Aβ_42_, anti-t-tau, anti- Aβ_40_, and anti-p-tau_181_) dissolved in 10 mM PBS. Each antibody solution was dropped on the sulfo-NHS-functionalized CNT channels and incubated overnight at 4 °C. To remove the unbound antibodies, the sensor chip was washed with 10 mM PBS containing Tween 20 (0.5 wt.%). Subsequently, we applied the mixture of 3 wt.% bovine serum albumin and 0.5 wt.% Tween 20 to each CNT device for 1.5 h at 4 °C.

### Preparation of biomarkers and procedure of biomarker sensing

We dissolved Aβ_42_ (1 mg) and Aβ_40_ (1 mg) in 480 μL of 1,1,1,3,3,3-hexafluoro-2-propanol and kept them overnight at room temperature. The solution was dispensed into Protein Lobind tube (Eppendorf AG, Germany) and vacuum-dried for 2 h. We dissolved human tau (100 μg) and synthetic p-tau_181_ (1 mg) in the deionized water. The solutions were aliquoted and stored at −20 °C for further experiments. We prepared biomarkers of femtomolar to picomolar concentrations by serially diluting the stock solution of each biomarker using a PBS solution (10 mM, pH 7.4). The stock concentration of each biomarker was 100 μg mL^−1^. We quantified the concentrations of biomarkers using an enzyme-linked immunosorbent assay (Invitrogen, Catalog Number KHB0041, Total Tau Human ELISA Kit) (Supplementary Fig. 35). We applied each biomarker to the bioreceptor-immobilized CNT device and incubated them for 15 min at the room temperature. For detecting of AD biomarker in human plasma, the blood plasma was diluted into 1/10. The resistance of the sensor chip was measured using a digital multimeter (Fluke 83 V, Fluke Co., USA).

### Clinical samples

The blood plasma samples of AD patients and healthy individuals were provided by the Biobank of Chungbuk and Chungnam National University Hospital, respectively, which are a member of the Korea Biobank Network. The clinical study was approved by the Ethics Committee and the Institutional Review Board (IRB) of the Korea Advanced Institute of Science and Technology (IRB-18–283) on 25 October 2018. All participants provided informed consent for the use of their specimens for research. The 10 μL of each patient sample was used for analysis.

### Measurement and characterization

We examined the morphology of as-synthesized CNT film using an atomic force microscope (AFM, Veeco Instruments, USA) and an S-4800 microscope (Hitachi High-technologies Co., Japan) at an accelerating voltage of 10 kV. We analyzed the UV-ozone-induced functionalization of CNT film using dispersive Raman spectroscope (Horiba Jobin-Yvon Ltd, France) and X-ray photoelectron spectroscope (Thermo VG Scientific, Inc., UK). In addition, we measured the fluorescence of fluorescein isothiocyanate-conjugated antibodies that were immobilized on CNTs using Victor 3 microplate reader (Perkin-Elmer, Inc., USA). For measuring transfer curves of liquid-gated aligned CNT-based sensor, PDMS wells were sealed on the top of the individual device to hold physiological buffers, in which an Ag/AgCl reference electrode was placed. All transfer curves were measured by a parameter analyzer (Keithley 4200A-SCS, Keithley Instruments, Inc., USA). We calculated the LOD and LOQ using the following equation: *κ* × *σ/S*, where *σ* is the residual SD of the linear regression, *S* is the slope of the regression line, and *κ* is the statistical confidence level. For determining the level of LOD and LOQ, the confidence levels were set as 3.3 and 10, respectively. The ROC analyses were performed using the MedCalc statistical software version 14.8.1 (MedCalc Software, Belgium).

### Reporting summary

Further information on research design is available in the Nature Research Reporting Summary linked to this article.

## Supplementary information

Supplementary Information

Reporting Summary

## Data Availability

All relevant data are available from the authors upon reasonable request. The source data underlying Figs. 2C-E, 3A-E, and 4A-G and Supplementary Figs. 4A-B, 6B, 7, 9B-C, 10,11,12,13B, 14A-D, 16D, 18A-B, 19A-B, 21, 22A-B, 23, 24, 25, 26, 27, 28A-L, 29, 32, 34, and 35 are provided as a Source Data file.

## Source data

Source Data

## Code availability

The software used for statistical analyses is available at http://www.medcalc.be/.
