# Supplementary material for: Clinically accurate diagnosis of Alzheimer’s disease via multiplexed sensing of core biomarkers in human plasma
Source: Nat Commun. 2020 Jan 8;11:119. doi: 10.1038/s41467-019-13901-z (PMC6949261; doi:10.1038/s41467-019-13901-z)
Supplement: Supplementary file 1 — Supplementary Information [file 41467_2019_13901_MOESM1_ESM.docx]

**Supplementary Information**

**Clinically Accurate Diagnosis of Alzheimer’s Disease via Multiplexed Sensing of Core Biomarkers in Human Plasma**

Kim et al.

**Supplementary Figures**


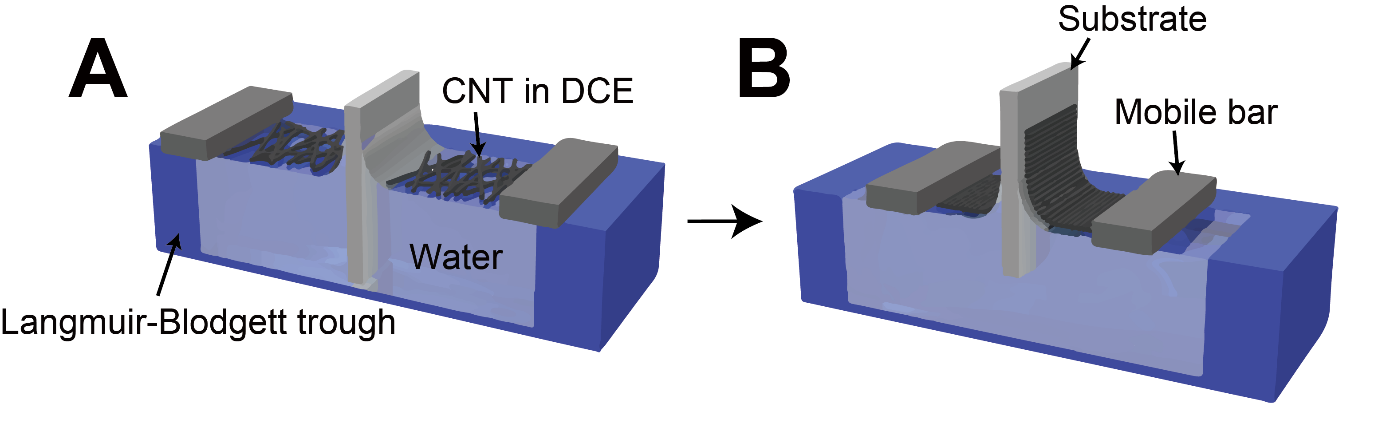


**Supplementary Figure 1.** Schematic illustration of Langmuir Blodgett assembly process. (A) CNTs in DCE solvent were floated on water sub-phase. (B) Mobile bars were used to apply a uniaxial compressive force into the CNTs. By repeating the compression-retraction LB cycling, the CNTs were assembled to the well-ordered array. The aligned CNTs were transferred into the SiO_2_/Si substrate by slowly pulling the substrate upward.

**
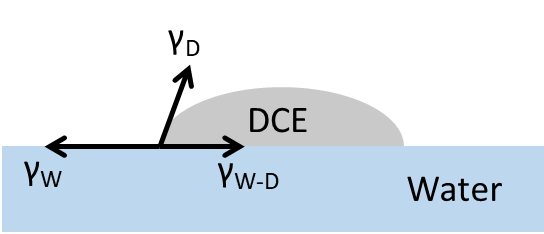
**

**Supplementary Figure 2.** Schematic illustration showing the surface tensions of water and DCE. As the DCE is non-polar solvent with a chemical formula of CH_2_Cl_2_, it is insoluble to water and spreads well on the surface of the water. Quantitatively, spreading coefficient (*S*) can be calculated as follows: *S = γ_W_ – (γ_D_ + γ_W-D_)*, where *γ_W_* (Surface tension of water) = 72.3 mN m^-1^, *γ_D_* (Surface tension of DCE)=28.2 mN m^-1^, and *γ_W-D_* (Surface tension between water and DCE)= 32.1 mN m^-1^. When the spreading coefficient is positive, a given solution will spread out on the water. The calculated spreading coefficient was +12.0 mN m^-1^, which indicated the spreading out of the PmPV-SWNT solution on water.


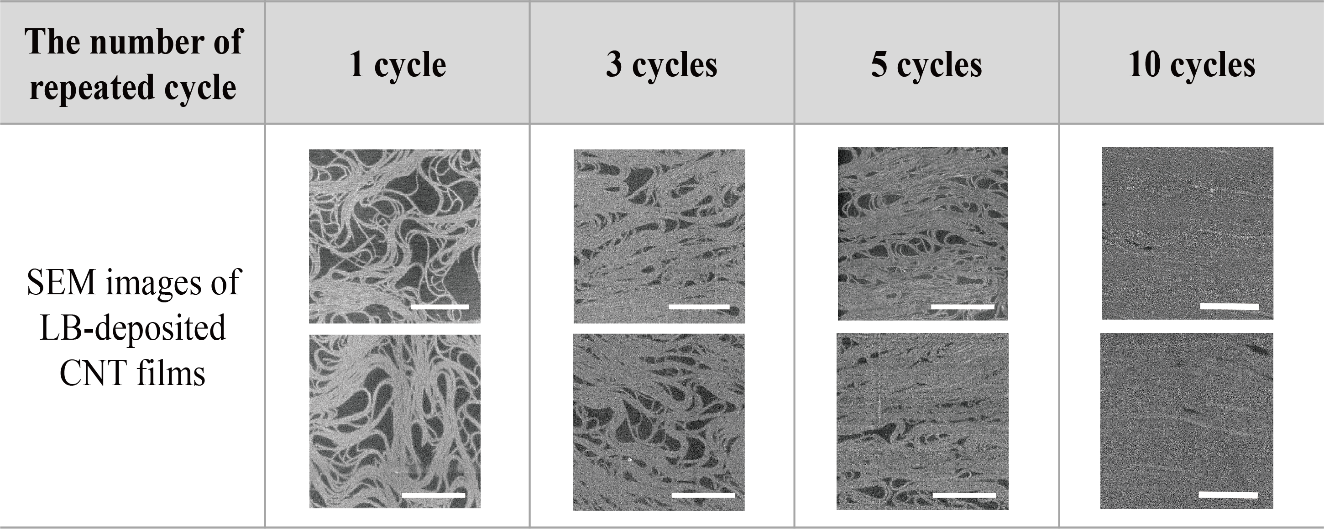


**Supplementary Figure 3**. SEM images of LB-transferred CNT films fabricated by different number of cycling at constant surface pressure (π = 35 mN m^-1^). LB-transferred CNT films fabricated by ten cycling exhibited highly packed and well-aligned CNTs whereas a large number of loops were observed in the CNT films prepared with lower cycling numbers. The scale bar represents 1 μm.


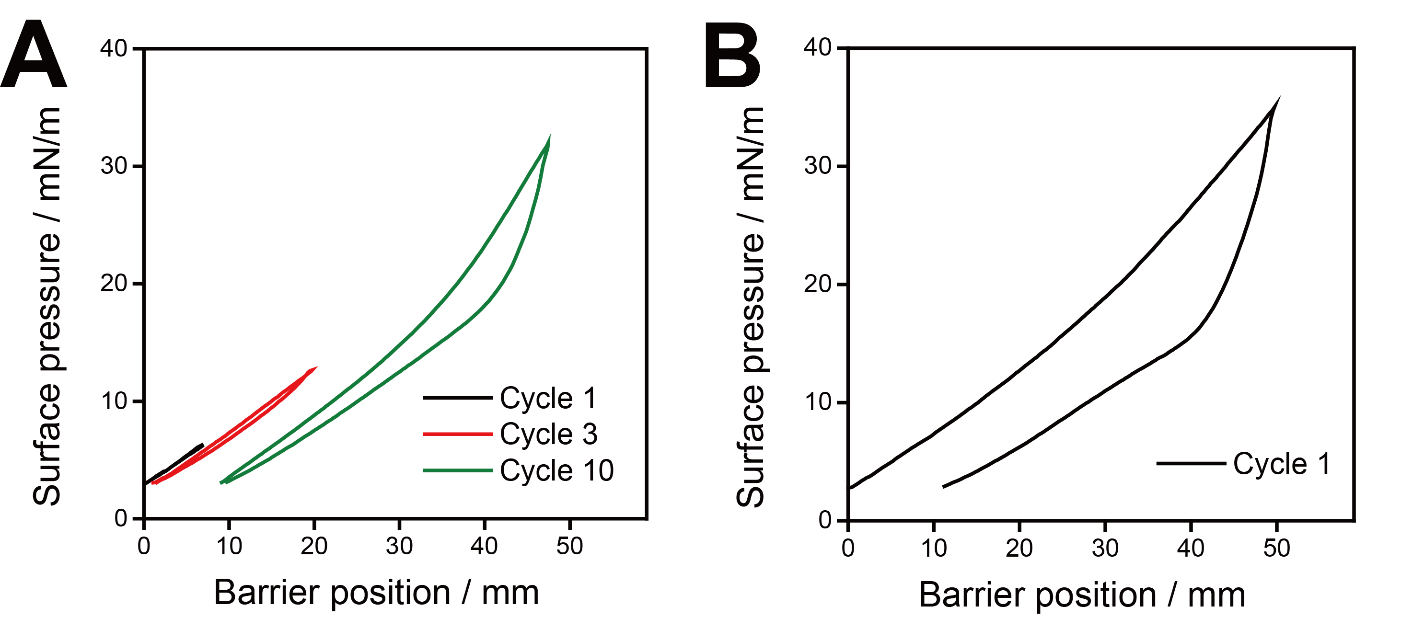


**Supplementary Figure 4**. Isotherm curves recorded during LB cycling. (A) Isotherm curves measured during the 10 cycle compression-retraction procedure. The target pressure of each cycle increases stepwise until reaching the surface pressure of 35 mN m^-1^, and the graph here shows the curves for the cycle of 1, 3, and 10, respectively. The isotherm curves of such repeated LB cycling showed negligible hysteresis. (B) Isotherm curve recorded during single LB cycling. The single cycle taken directly up to the surface pressure of 35 mN m^-1^ exhibited a large hysteresis. Source data are provided as a Source Data file.


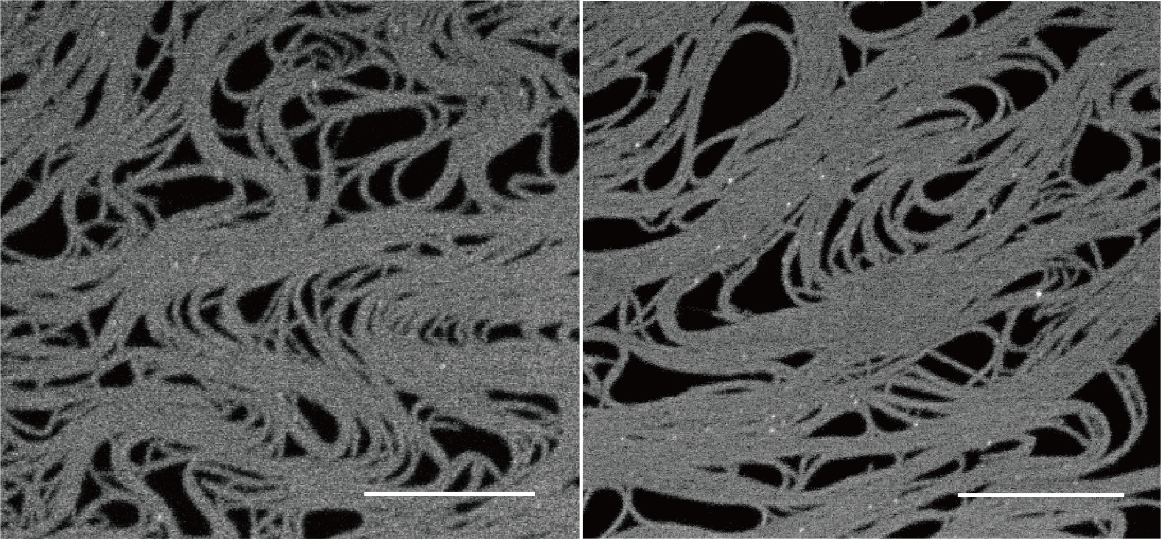


**Supplementary Figure 5**. SEM images of CNT films transferred after single LB cycling. In comparison to the CNT films fabricated using repeated LB cycling, a large number of loops were observed in CNT films. The scale bars represent 500 nm. Source data are provided as a Source Data file.


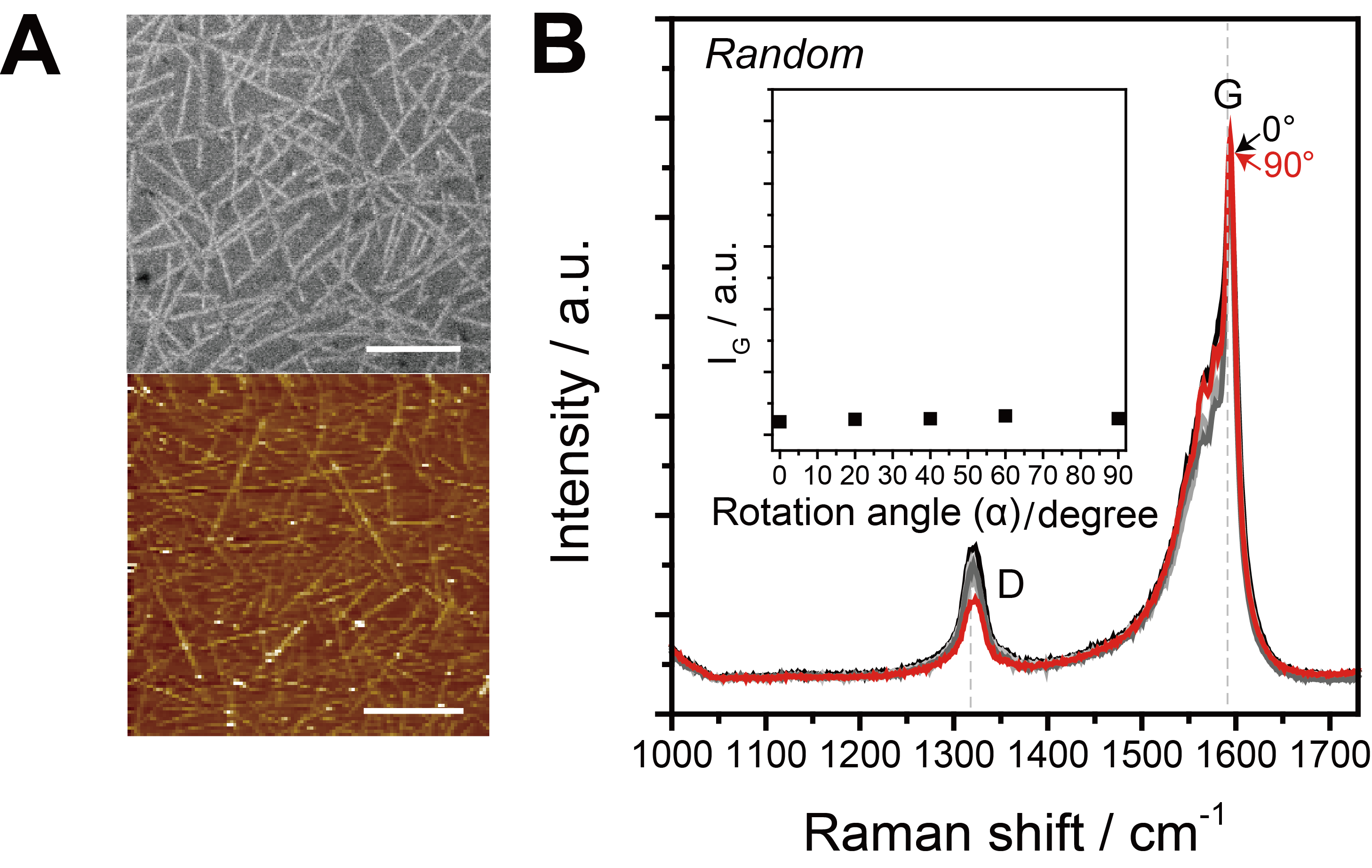


**Supplementary Figure 6**. Characterization of the random-network CNT films. (A) SEM and AFM images of Random-network CNT film that was prepared using conventional spin-coating method. When fabricating the random-network CNT films, we used the same CNT solutions with that of the densely aligned CNT films. The scale bars in SEM and AFM images indicate 250 nm and 500 nm, respectively. (B) Polarized Raman spectra of the random-network CNT film recorded at various angles between 633 nm incident laser and direction of CNT film. The inset shows the angular dependence of the Raman intensity at 1595 cm^-1^. It had no tendency of the Raman intensity with increasing the rotation angle. Source data are provided as a Source Data file.


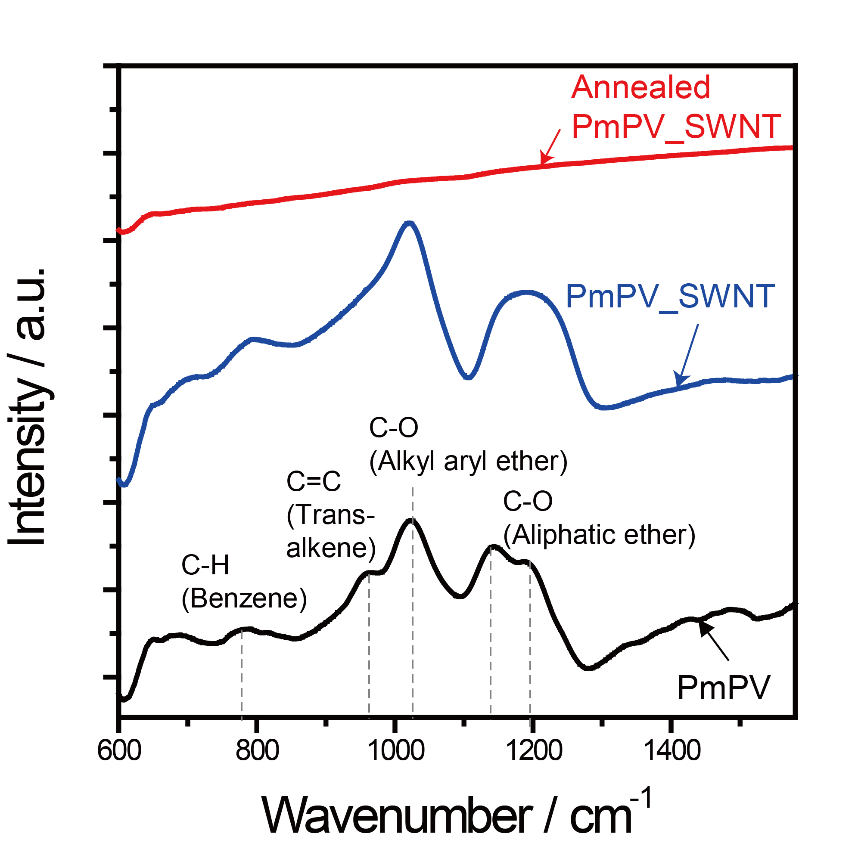


**Supplementary Figure 7.** FTIR spectra of PmPV and PmPV-wrapped CNT film before and after heat treatment. Each peak represents characteristic functional groups in the PmPV polymer structure. The PmPV polymer was effectively removed by thermal annealing in Ar atmosphere. Source data are provided as a Source Data file.


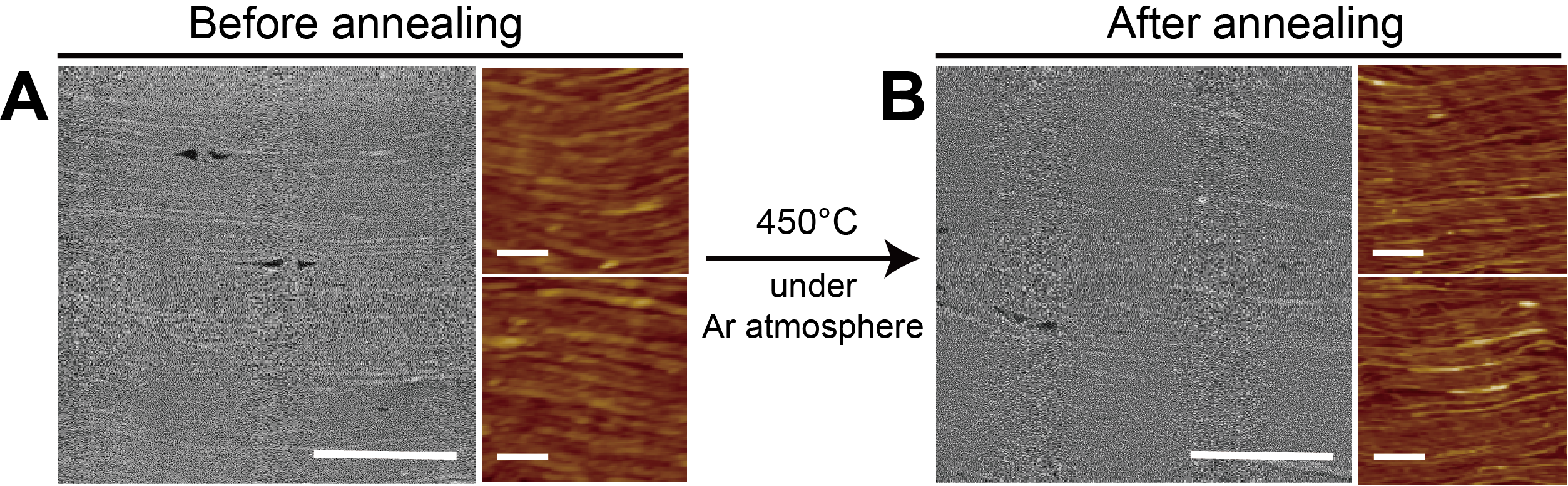


**Supplementary Figure 8**. SEM and AFM images of densely aligned CNT film (A) before and (B) after annealing (450 °C, under Ar atmosphere). The thermal annealing did not cause curling or rolling of LB-deposited CNTs. The scale bars in SEM and AFM images indicate 1 μm and 500 nm, respectively.

**
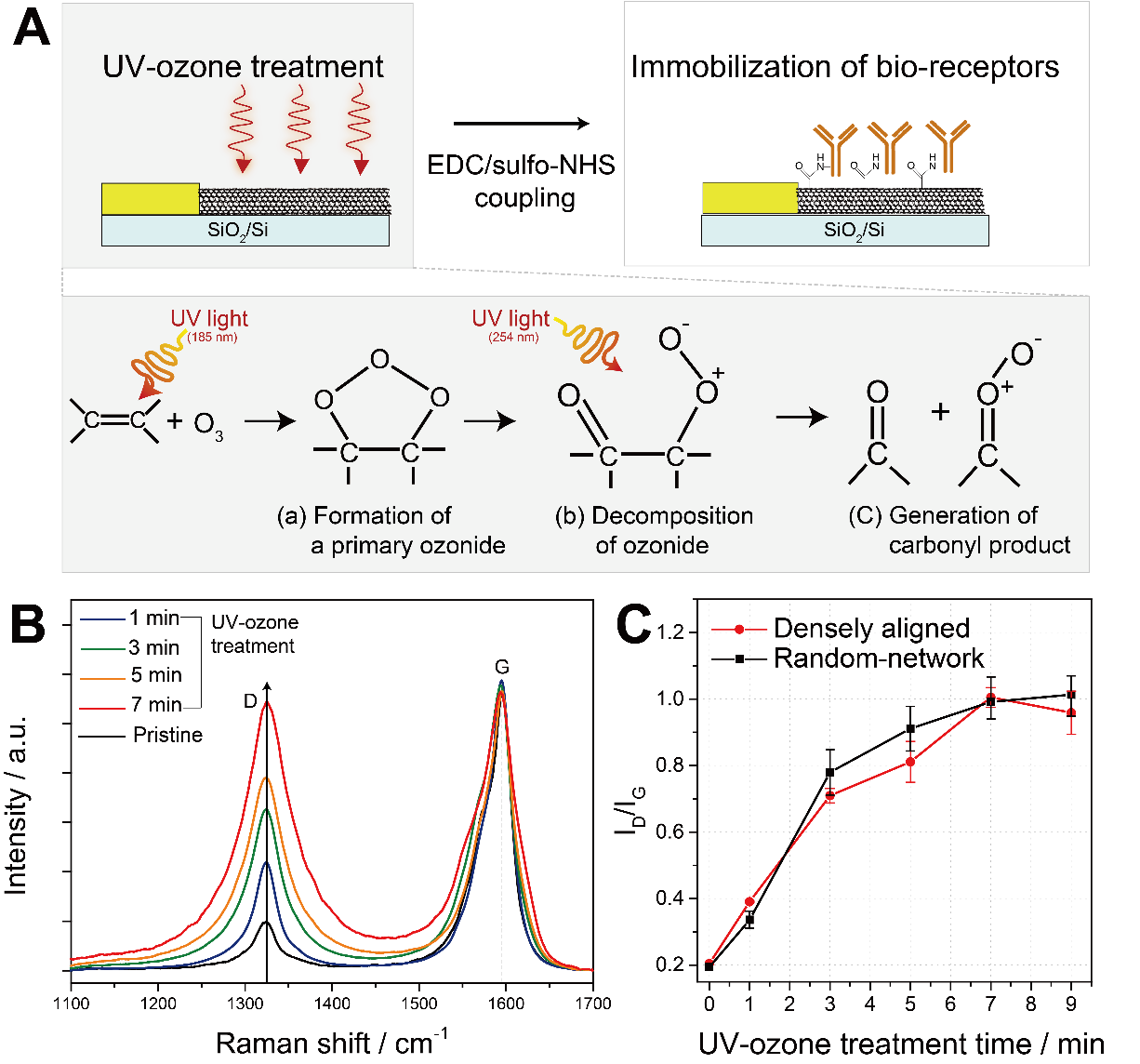
**

**Supplementary Figure 9.** Functionalization of CNTs with bio-receptors. (A) Schematic illustration showing the procedure of modifying the surface of CNTs with bio-receptors. Prior to the conjugation, the surface of CNTs was functionalized with carboxyl groups by UV-ozone treatment. The UV-ozone-induced breakage of sp^2^ C-C bonds on CNT sidewalls occurs through Criegee’s mechanism. The exposure of CNTs to UV light (185 nm) generates ozone molecules that chemisorb on the sidewalls of the CNTs via a [2+3] cycloaddition. Furthermore, the UV light of 254 nm dissociates the ozone molecules, spontaneously generating carbonyl groups on the CNTs. The illustration of Criegee’s mechanism was reproduced from the following references with minor modifications (*J. Phys. Chem. B* 2002, **106**, 2136; *Angew. Chem., Int. Ed.* 1975, **14**, 745). (B) Change in the Raman spectra of the CNTs with increasing the degree of UV-ozone-induced oxidation. Raman spectroscopy showed the two characteristic peaks of CNT, i.e., D and G bands at 1325 and 1595 cm^-1^, respectively. (C) Changes in the ratio of I_D_ over I_G_ of the densely aligned or random network CNT film during the UV-ozone induced oxidation. The values of I_D_/I_G_ were gradually increased with increasing the UV-ozone treatment time. All the values represent the mean ± standard deviation (n=3). Source data are provided as a Source Data file.


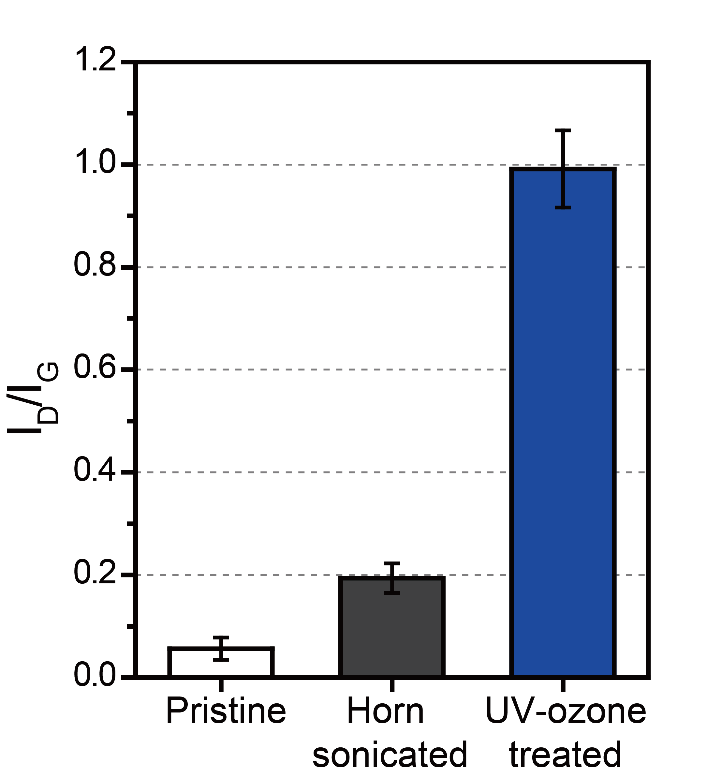


**Supplementary Figure 10.** Comparison of I_D_ to I_G_ ratio of pristine, horn sonicated, and UV-ozone treated CNT films. As a pre-step of the UV-ozone-induced functionalization, we conducted horn-sonication for 1 h at 70% amplitude to disperse CNTs in the DCE solvent. The horn sonication increased the defect ratio of CNTs but to a much lesser degree than the UV-ozone-treated CNTs; the average I_D_/I_G_ of UV-ozone treated CNT films was ~1.0 whereas that of the horn-sonicated CNT film was 0.19. The UV-ozone was exposed to CNTs for 7 min. All the values represent the mean ± standard deviation (n=3). Source data are provided as a Source Data file.


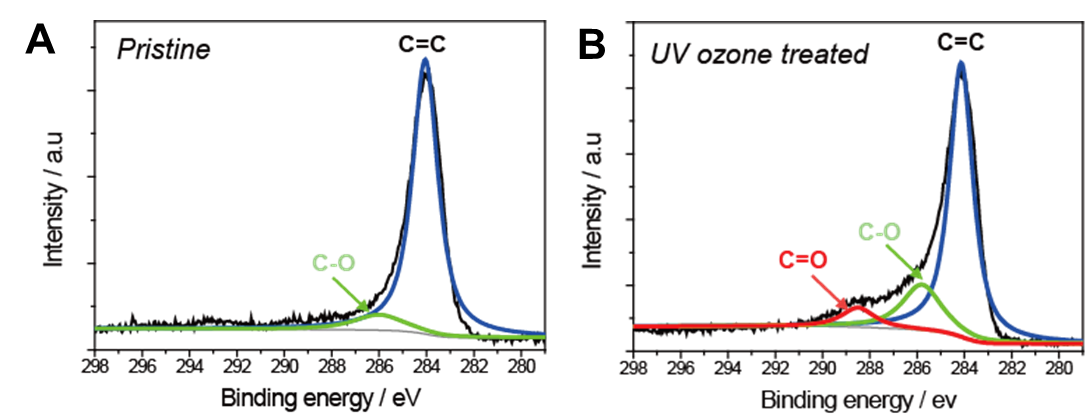


**Supplementary Figure 11.** X-ray photoelectron spectroscopic (XPS) spectra of the densely aligned CNT film (A) before and (B) after UV-ozone treatment. After UV-ozone-induced oxidation of CNTs, the XPS peak assigned to C=O newly appeared and the content of oxygen molecules on the CNT surface gradually increased from 7.4% to 16.9%. Source data are provided as a Source Data file.


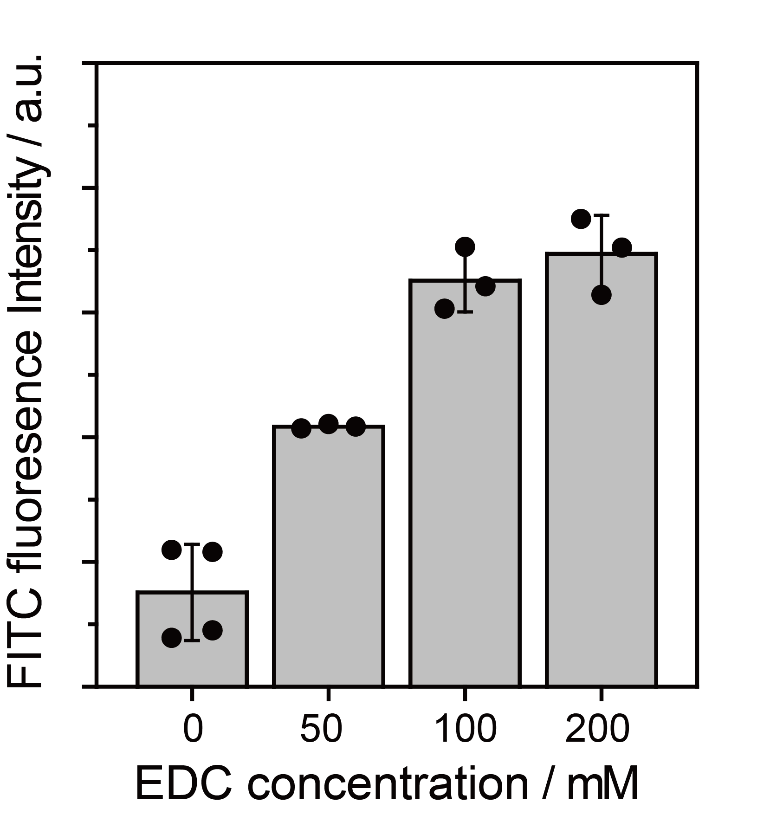


**Supplementary Figure 12.** Fluorescence intensity of fluorescein isothiocyanate (FITC)-labeled anti-IgG molecules, which was covalently conjugated on the CNT surface (λ_ex_= 490 nm, λ_em_=525 nm). The carboxyl groups-functionalized CNT films were conjugated with FITC-labeled anti-IgG using the carbodiimide crosslinker. The successful conjugation was confirmed by the increase in fluorescence intensity with increasing the concentration of EDC and sulfo-NHS. The concentration ratio of EDC:sulfo-NHS was 1:2.5. All the values represent the mean ± standard deviation (n=3). Source data are provided as a Source Data file.

**
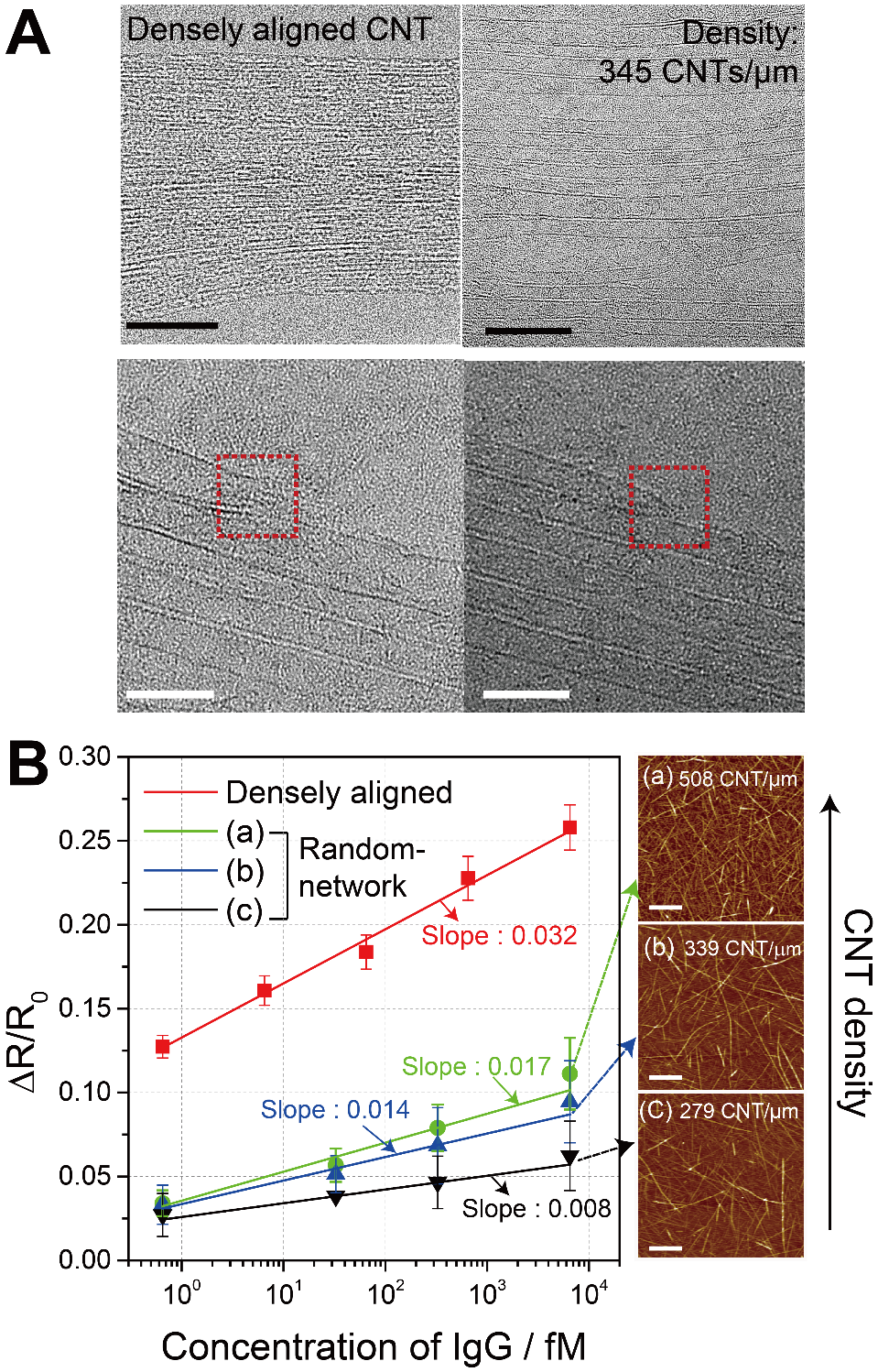
**

**Supplementary Figure 13.** Sensing performance of densely aligned CNT device in comparison to random network CNT devices having different densities (i.e., 279, 339, and 508 CNTs/μm). (A) Top-view TEM images of the densely aligned CNT film. We calculated the average line density by analyzing 7 TEM images and 24 lines. The red squares in the TEM images indicate the ends of individual CNTs. The scale bars of upper left and right images are 25 nm and 10 nm, respectively. The bottom TEM images’ scale bars are 5 nm. (B) Changes in the resistances of densely aligned and random-network CNT sensor arrays upon exposure to IgG. The densely aligned CNT array (345 CNTs μm^-1^) exhibited more than two fold higher sensitivity than random-network arrays with similar (339 CNTs μm^-1^) or higher densities (508 CNTs μm^-1^). All the values represent the mean ± standard deviation (n=3). The scale bars in AFM images are 1 μm. Source data are provided as a Source Data file.

**
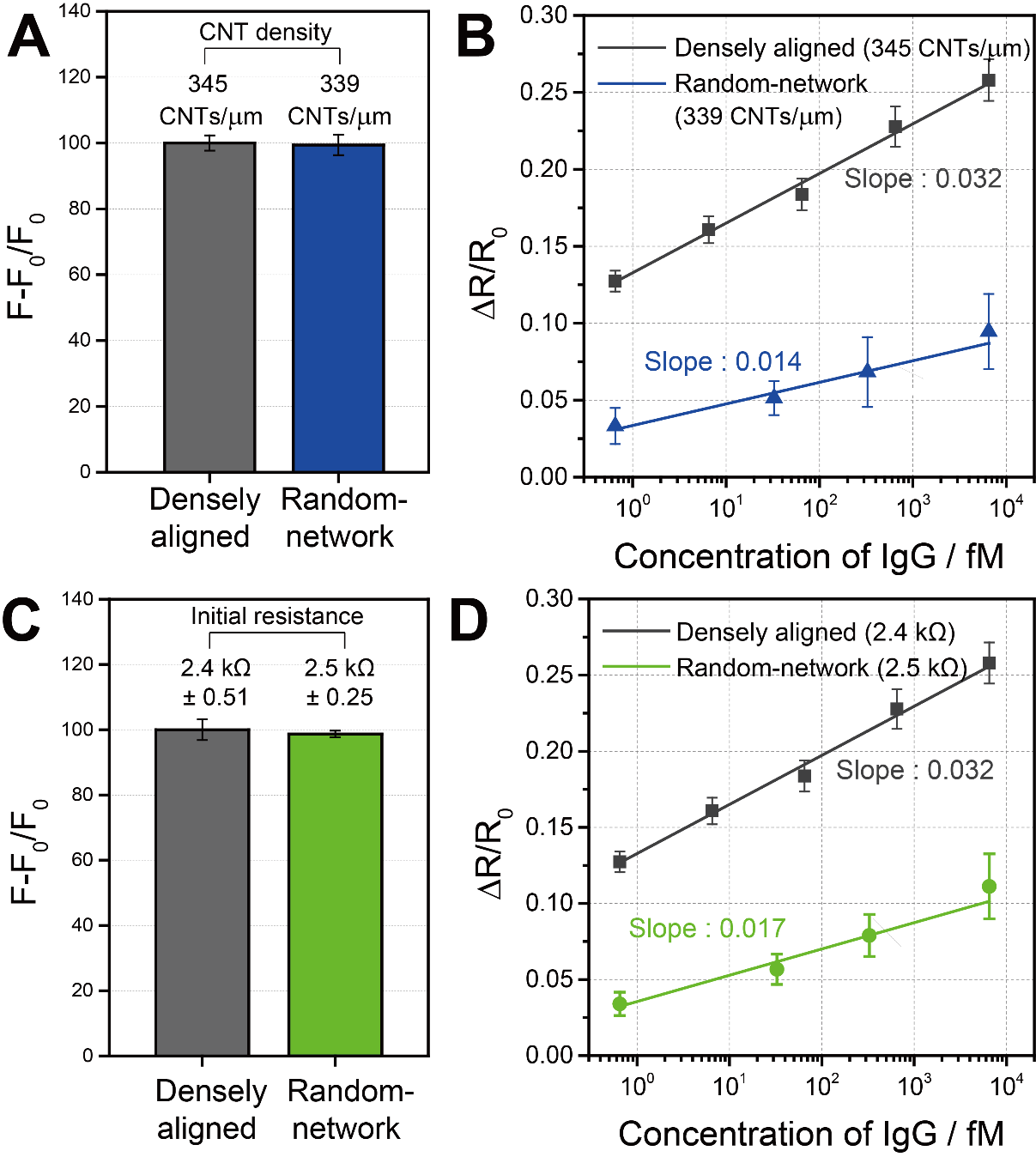
**

**Supplementary Figure 14.** The amount of anti-IgG and the sensitivities of the densely aligned CNT sensor arrays in comparison to the random-network CNT arrays having (**A**, **B**) similar CNT densities or (**C, D**) comparable initial resistance. Note that the CNTs’ lengths are similar for all of the arrays because we used non-destructive thin-film formation methods (that do not affect the CNT length) and the same CNT solutions. The fluorescence of FITC-labeled anti-IgG was measured at 525 nm (λ_ex_= 490 nm). All the values represent the mean ± standard deviation (n=3). Source data are provided as a Source Data file.

**
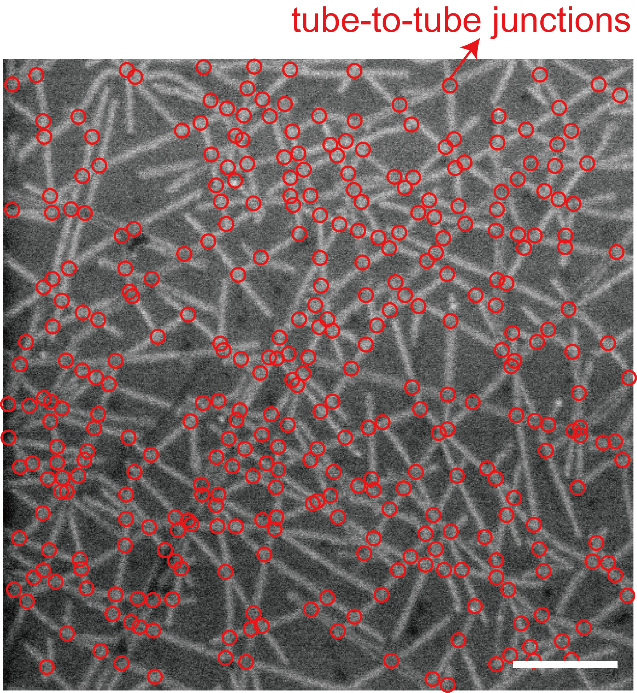
**

**Supplementary Figure 15.** SEM images of random-network CNT films. The tube-to-tube junctions are indicated by red circles. The random-network CNT film has approximately 134.57 tube-to-tube junctions μm^-2^ (scale bar: 250 nm).


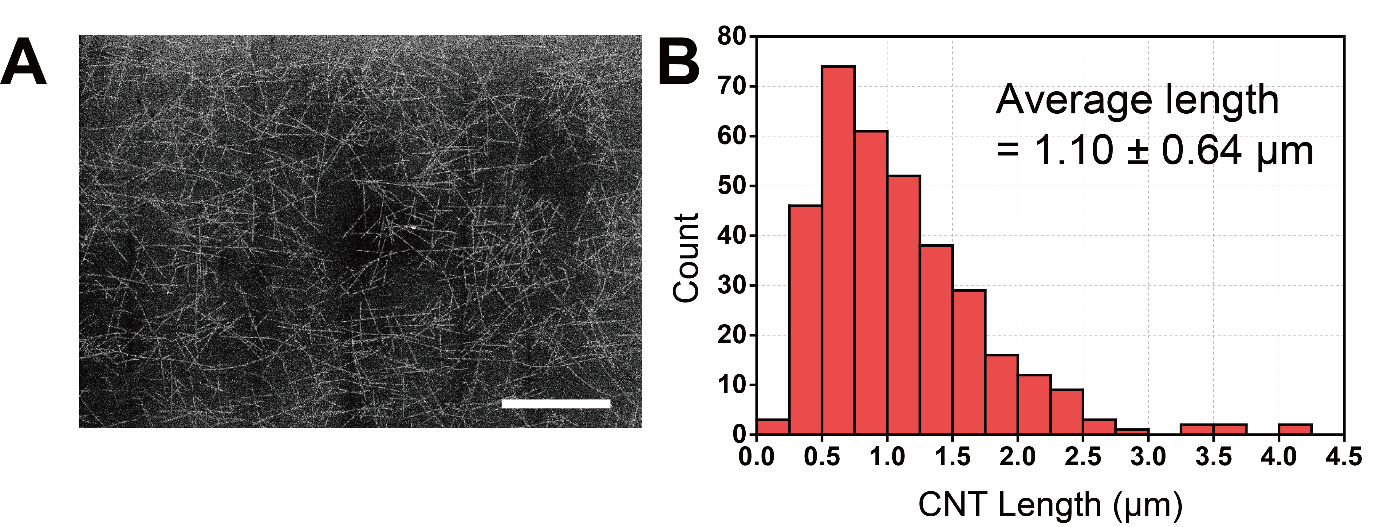


**Supplementary Figure 16**. The average lengths of CNTs. (A) SEM images of a random-network CNT film. The scale bars represent 2.5 μm. (B) The lengths of 350 CNTs in the SEM image. The average length of CNTs is 1.10 ± 0.64 µm. Source data are provided as a Source Data file.


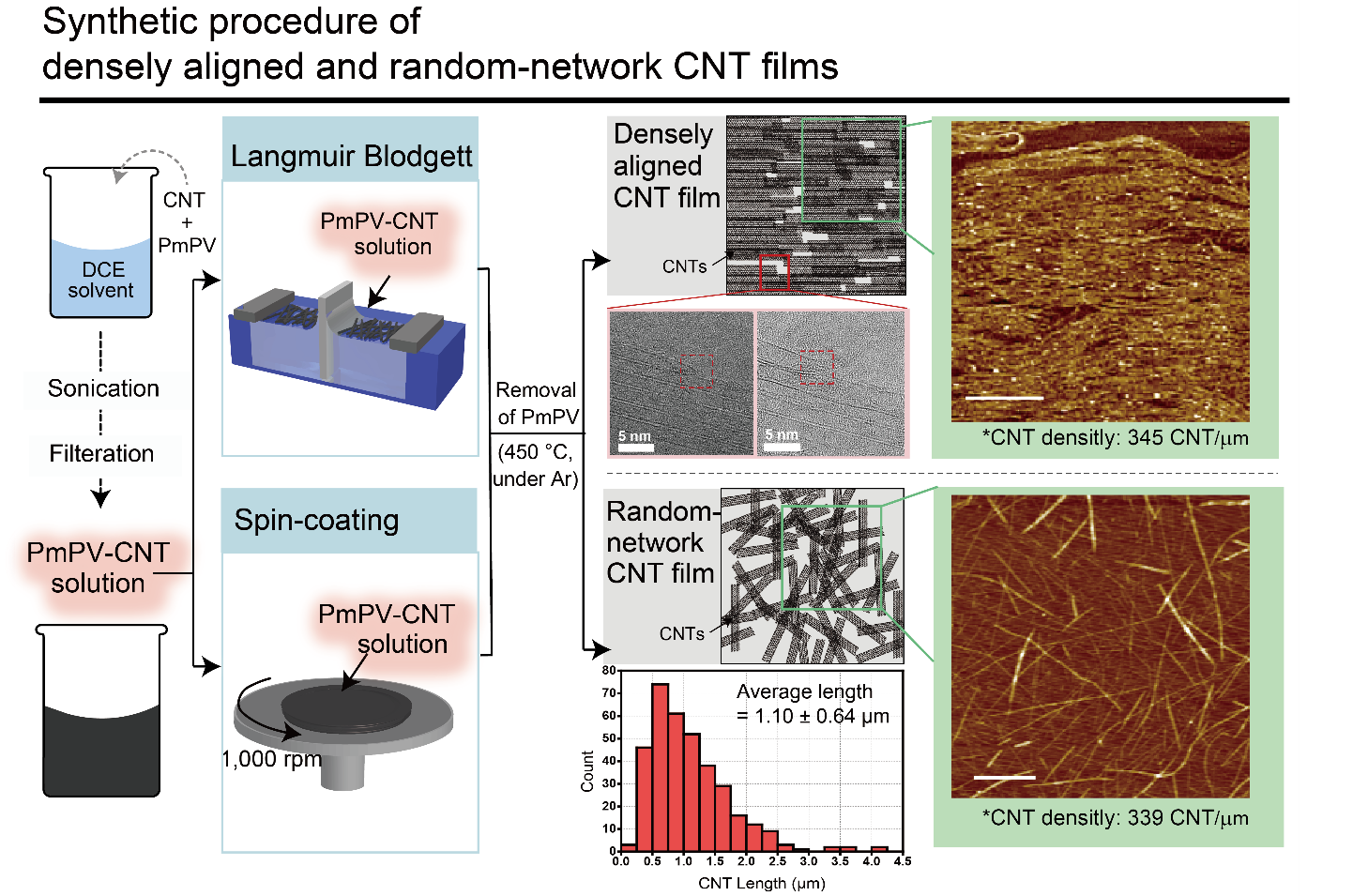


**Supplementary Figure 17.** Schematic illustration showing the thin-film formation procedure of densely aligned and random-network CNT films. Because we used the same CNT source (OCSiAL Co., USA) and the same CNT solution, and the entire procedure (i.e., spin-coating method for random-network CNTs and Langmuir Blodgett transfer for the densely aligned CNTs) is handled under a mild condition, the lengths of CNTs in the random-network film are expected to be the same to those in the LB-transferred film. The scale bars in AFM images of the densely aligned and random-network CNT films indicate 500 nm and 1 μm, respectively.


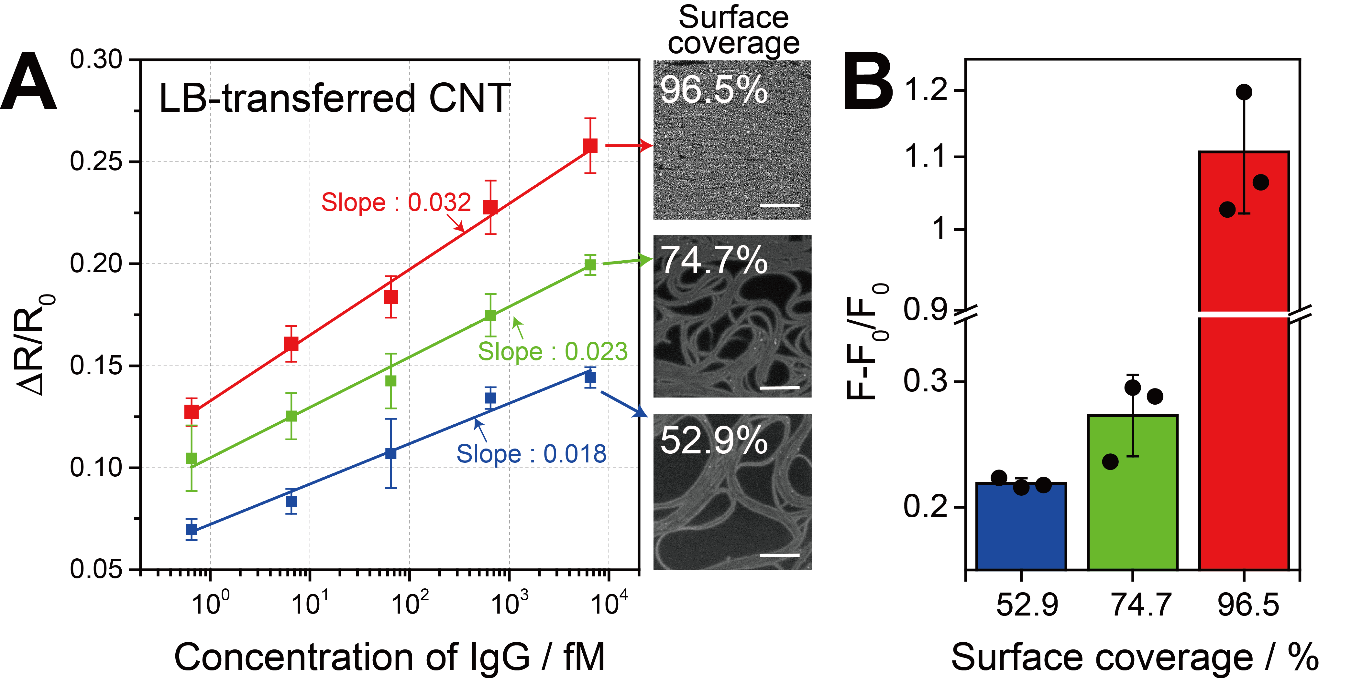


**Supplementary Figure 18.** Comparison of sensing performances of LB-transferred CNT-based sensor arrays having different surface coverage of CNTs. (A) Changes in resistance of the LB-transferred CNT sensor arrays having the different surface coverages. We estimated the surface coverages of CNTs using automated image analysis. For each data point, different set of devices was used. Data reproducibility was confirmed by two additional experiments. All the values represent the mean ± standard deviation. Note that we fixed the surface target pressure at 35 mN m^-1^ to maintain the CNT’s surface coverage at 96.5% throughout this study (scale bar: 250 nm) (B) Fluorescence intensity of FITC-conjugated anti-IgG immobilized on CNT films. The fluorescence of FITC-labeled anti-IgG was measured at 525 nm (λ_ex_= 490 nm). Data reproducibility was confirmed by two additional experiments. All the values represent the mean ± standard deviation. Source data are provided as a Source Data file.


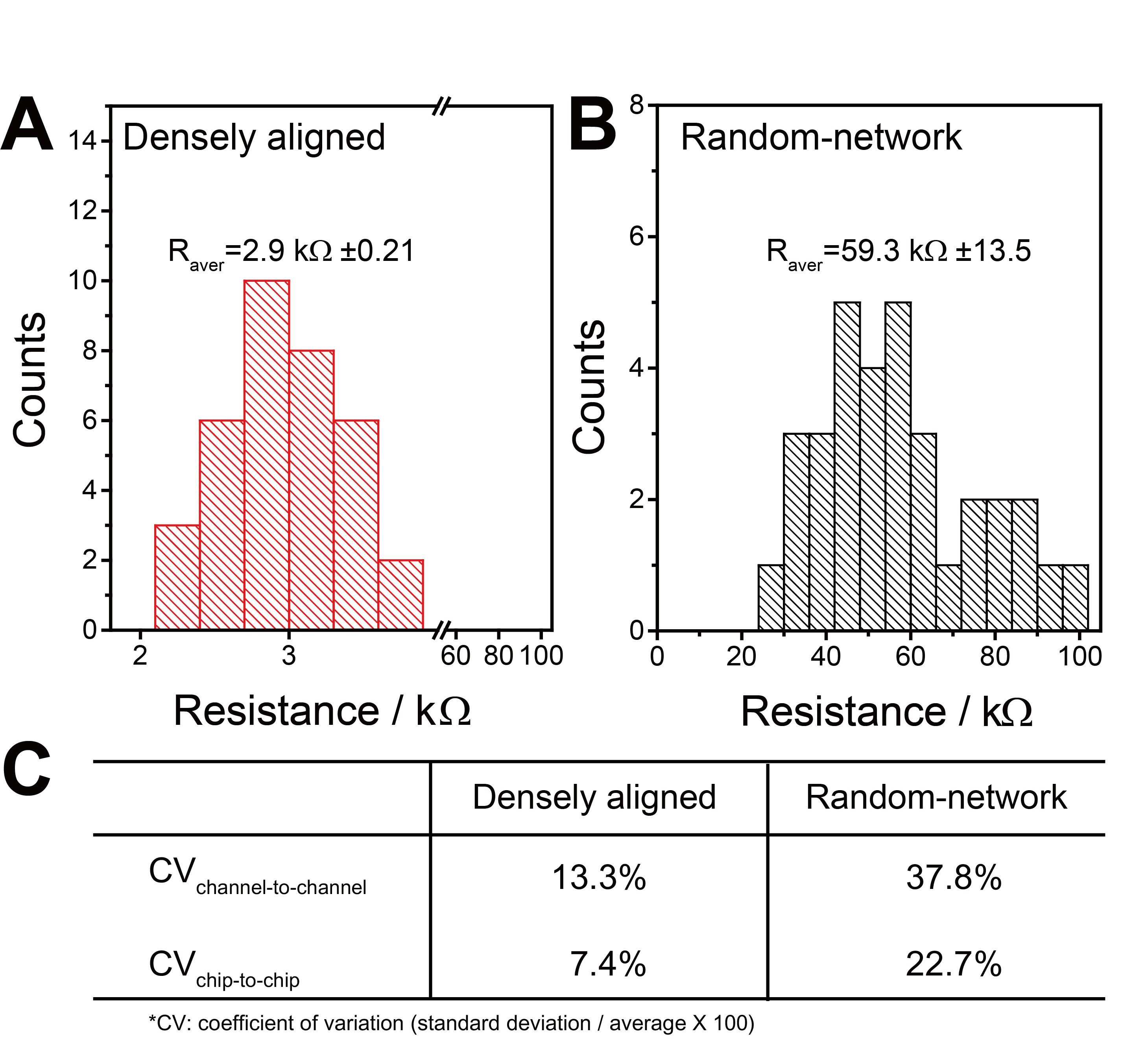


**Supplementary Figure 19**. Histogram showing the resistances of (A) densely aligned or (B) random-network CNT device. The densely aligned CNT sensor array showed 20.4 times lower resistance than that of random-network CNT device array. (C) The coefficient of variation (CV) for the resistance of the densely aligned CNT devices and arrays were 13.3% and 7.4%, respectively, which were 2-3 times lower than that of random-network CNT device and arrays. Source data are provided as a Source Data file.

**
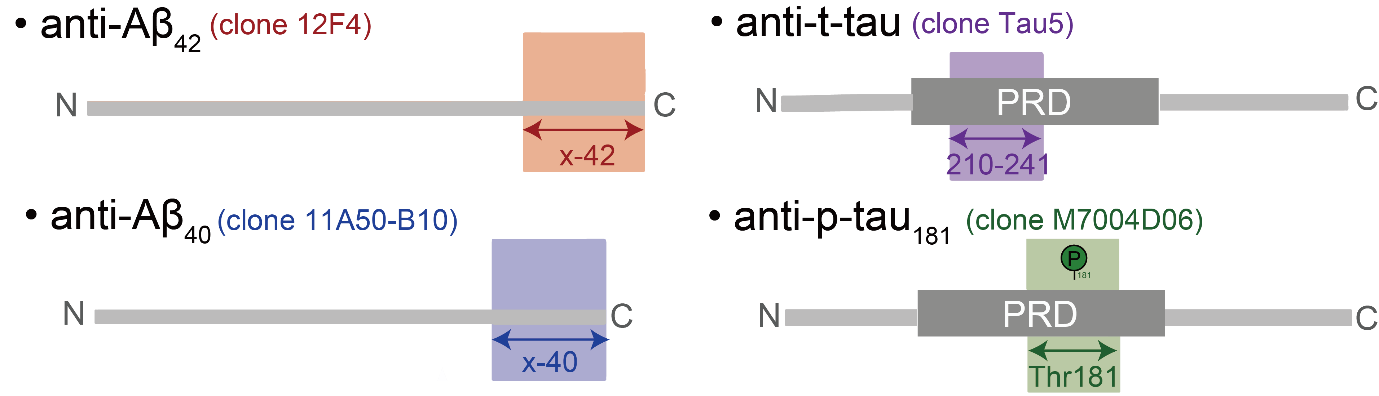
**

**Supplementary Figure 20.** Antibodies used in the densely aligned CNT sensor array. Each antibody binds to specific sequence of the corresponding biomarker; the 12F4 and 11A50-B10 antibodies recognize the C-terminus of Aβ_42_ and Aβ_40_, respectively. The epitope of Tau5 lies within amino acids 210-241 of tau proteins. The M7004D06 antibody is reactive to the human tau phosphorylated at 181 residue.


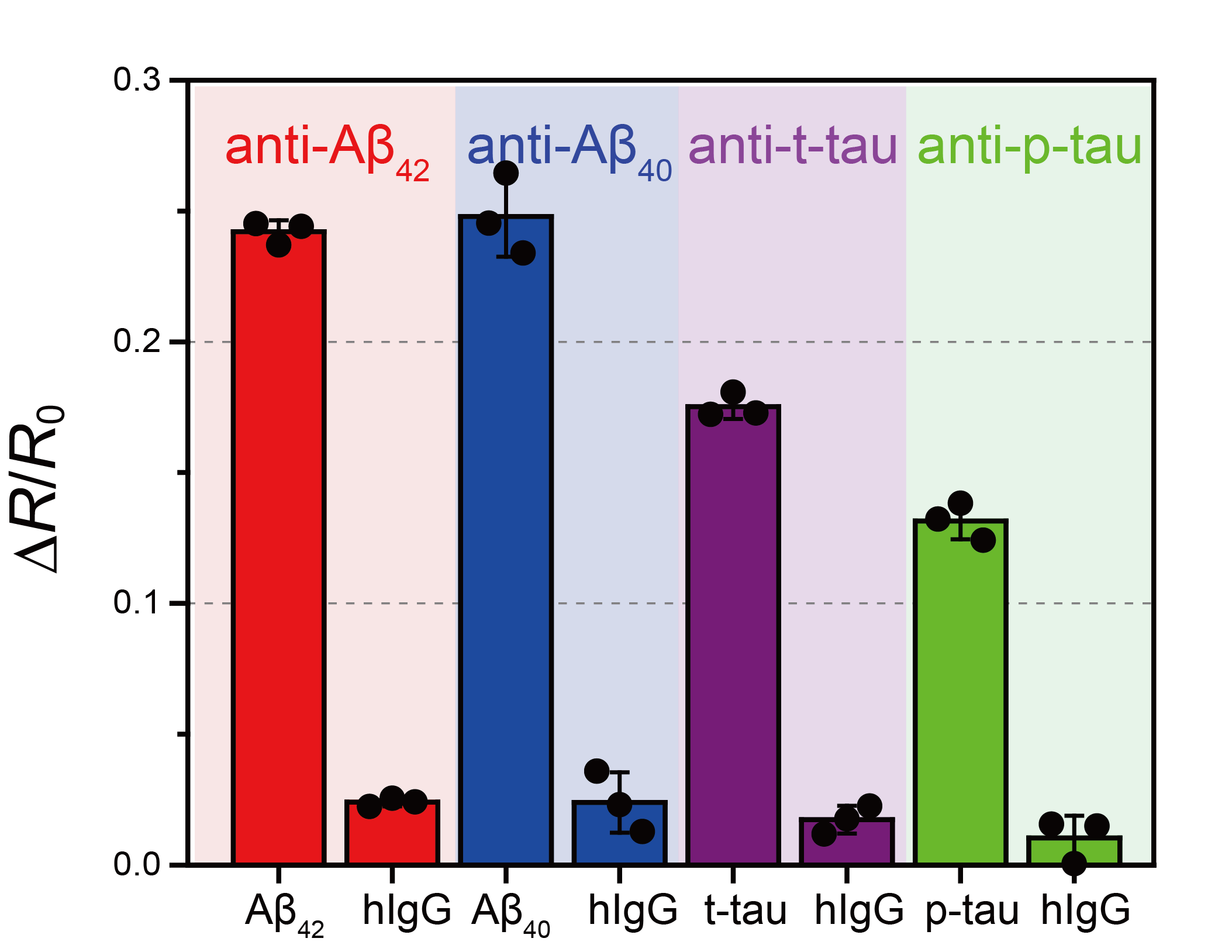


**Supplementary Figure 21**. Changes in the resistance of the densely aligned CNT sensor array upon the addition of AD biomarkers and human IgG (hIgG, negative controls). The concentrations of Aβ_42_ and Aβ_40_ were 2.22×10^6^ fM, 2.31×10^6^ fM, respectively. In the case of t-tau and p-tau, the concentrations were 2.18×10^5^ fM and 3.57×10^6^ fM, respectively. The human IgG’s concentration was 6.55×10^6^ fM. The measurement was performed in triplicate and all reported values represent the mean ± standard deviation. Source data are provided as a Source Data file.

**
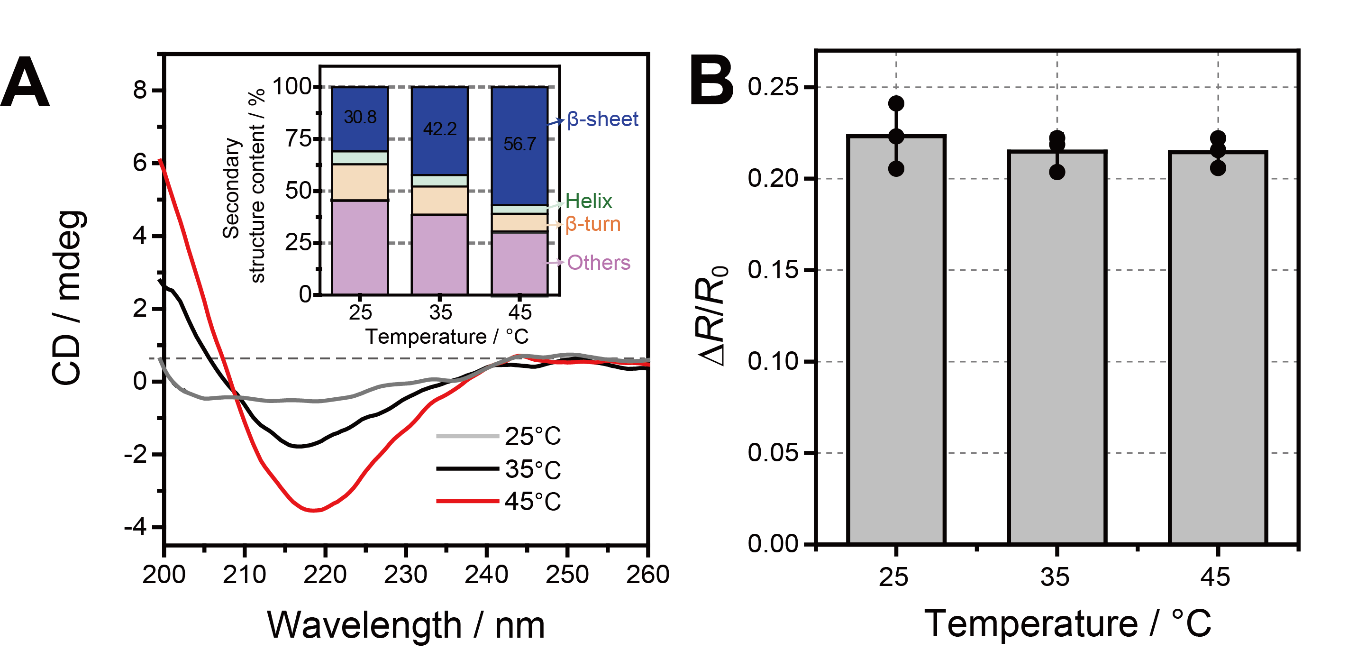
**

**Supplementary Figure 22**. Effect of biomarker’s thermal denaturation on the sensing response of the densely aligned CNT sensor array. (A) Structural changes of Aβ_42_ with the increasing temperature from 25^◦^C to 45^◦^C. Intense peaks at 200 nm and 216 nm in CD spectra were observed after thermal denaturation. These results indicate the conformational changes of Aβ_42_’s native structure into β-sheet-rich secondary structure. (B) Changes in the resistance of the densely aligned CNT sensor array upon exposure to thermally denatured Aβ_42_ at different temperature. All the values represent the mean ± standard deviation (n=3). Source data are provided as a Source Data file.


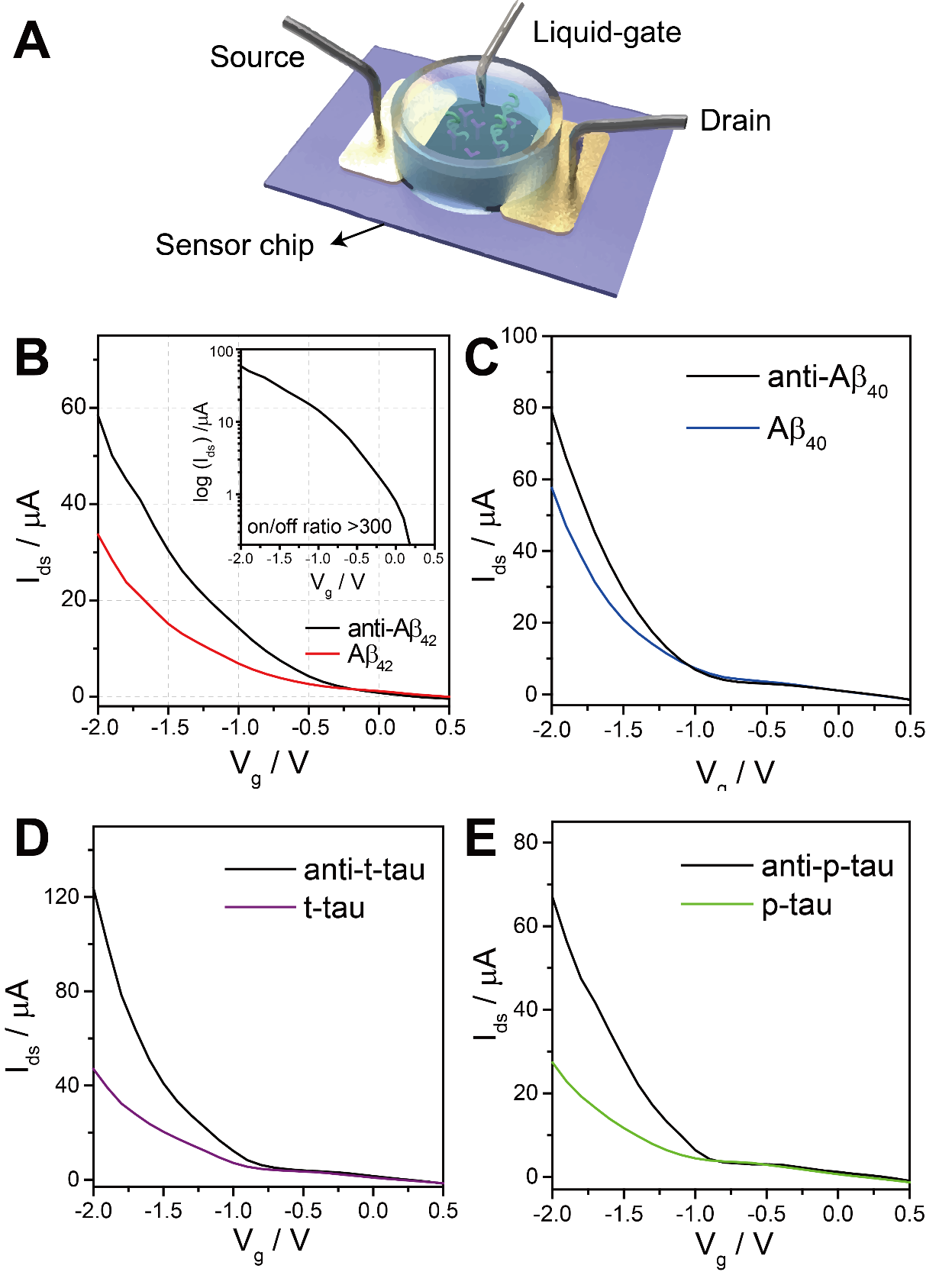


**Supplementary Figure 23**. Transfer curves of the densely aligned CNT sensor arrays. (A) Schematic illustration of densely aligned CNT device with a liquid-ion gate. We used the Ag/AgCl reference electrode as a gate electrode and 1 μM PBS buffer (pH 7.4) as an electrolyte. Transfer curves of the sensor array upon exposure to (B) Aβ_42_, (C) Aβ_40_, (D) t-tau, and (E) p-tau at a constant V_ds_ = -10 mV. The on/off ratio of densely aligned CNT sensor array was approximately 10^3^. Source data are provided as a Source Data file.


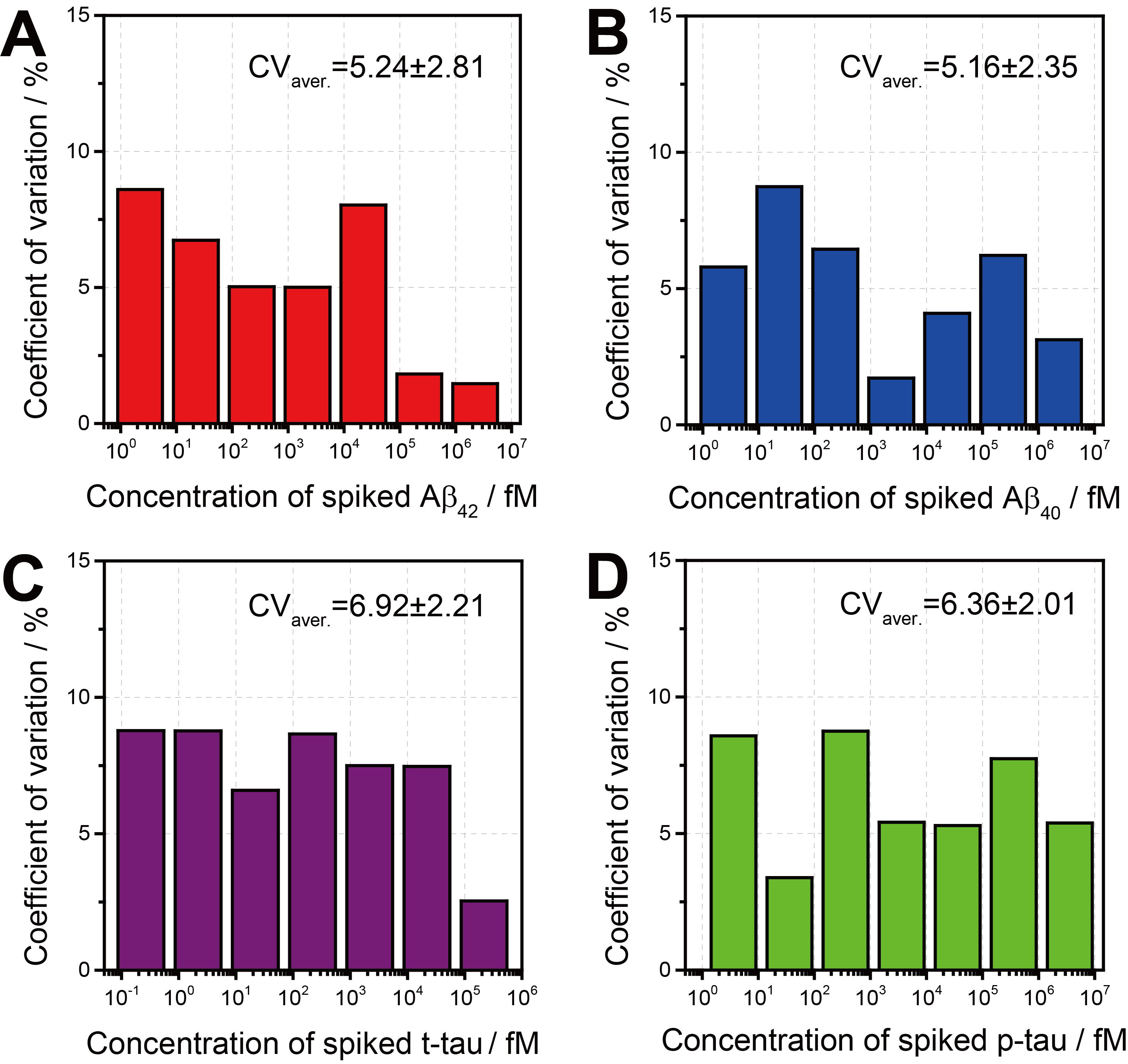


**Supplementary Figure 24**. The coefficient of variation (CV) for the resistance changes of the densely aligned CNT sensor array upon the exposure to (A) Aβ_42_, (B) Aβ_40_, (C) t-tau, and (D) p-tau. The values of CV in all cases were under 10%, which indicates that the densely aligned CNT sensor array had a high degree of precision in detecting AD biomarkers. Source data are provided as a Source Data file.


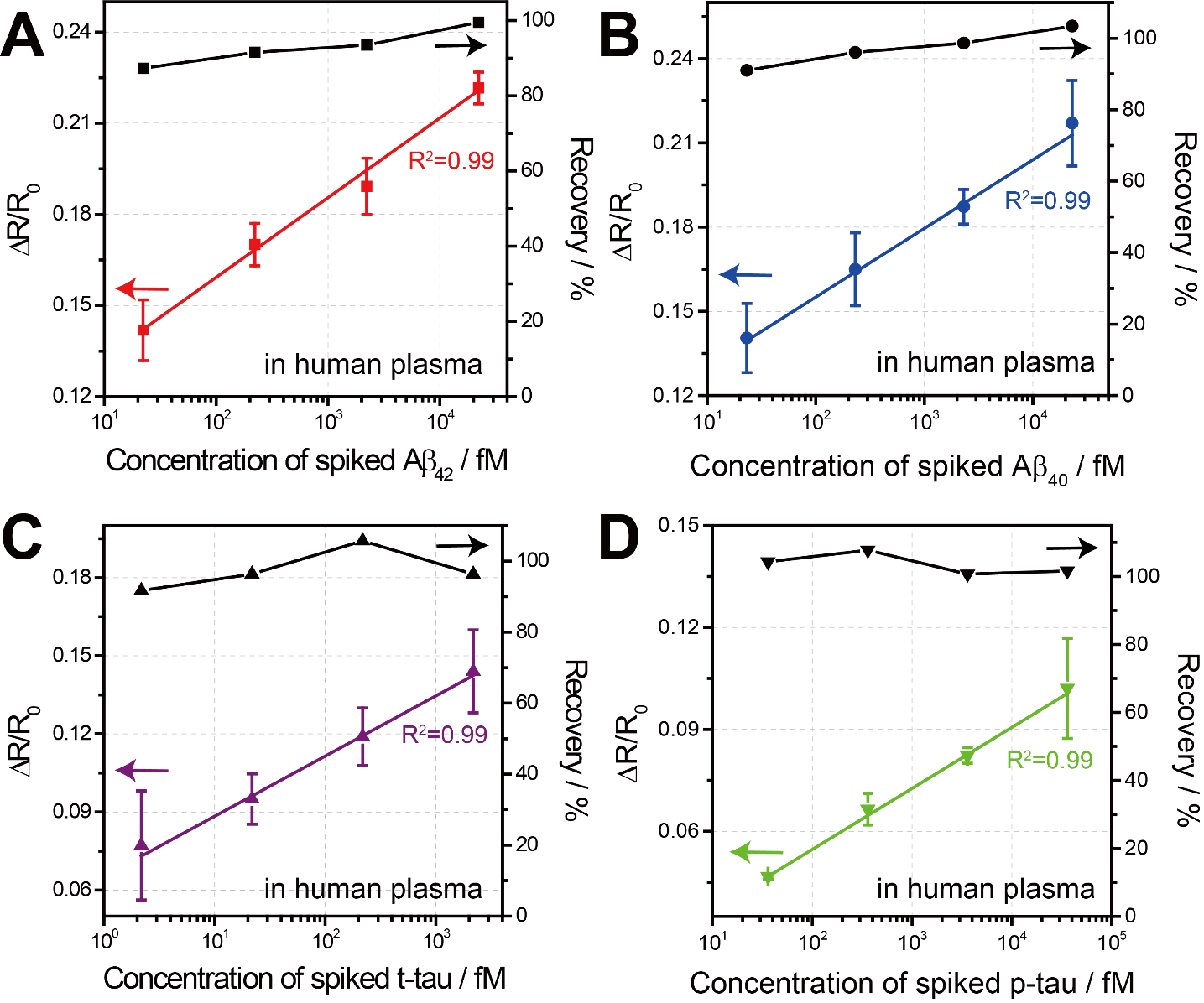


**Supplementary Figure 25**. Changes in resistance of the densely aligned CNT sensor array with increasing the concentrations of the spiked (A) Aβ_42_, (B) Aβ_40_, (C) t-tau and (D) p-tau in human plasma. Each data point was attained using a different set of devices. The data reproducibility was confirmed by two additional experiments. All reported values represent the mean ± standard deviation. Source data are provided as a Source Data file.

**
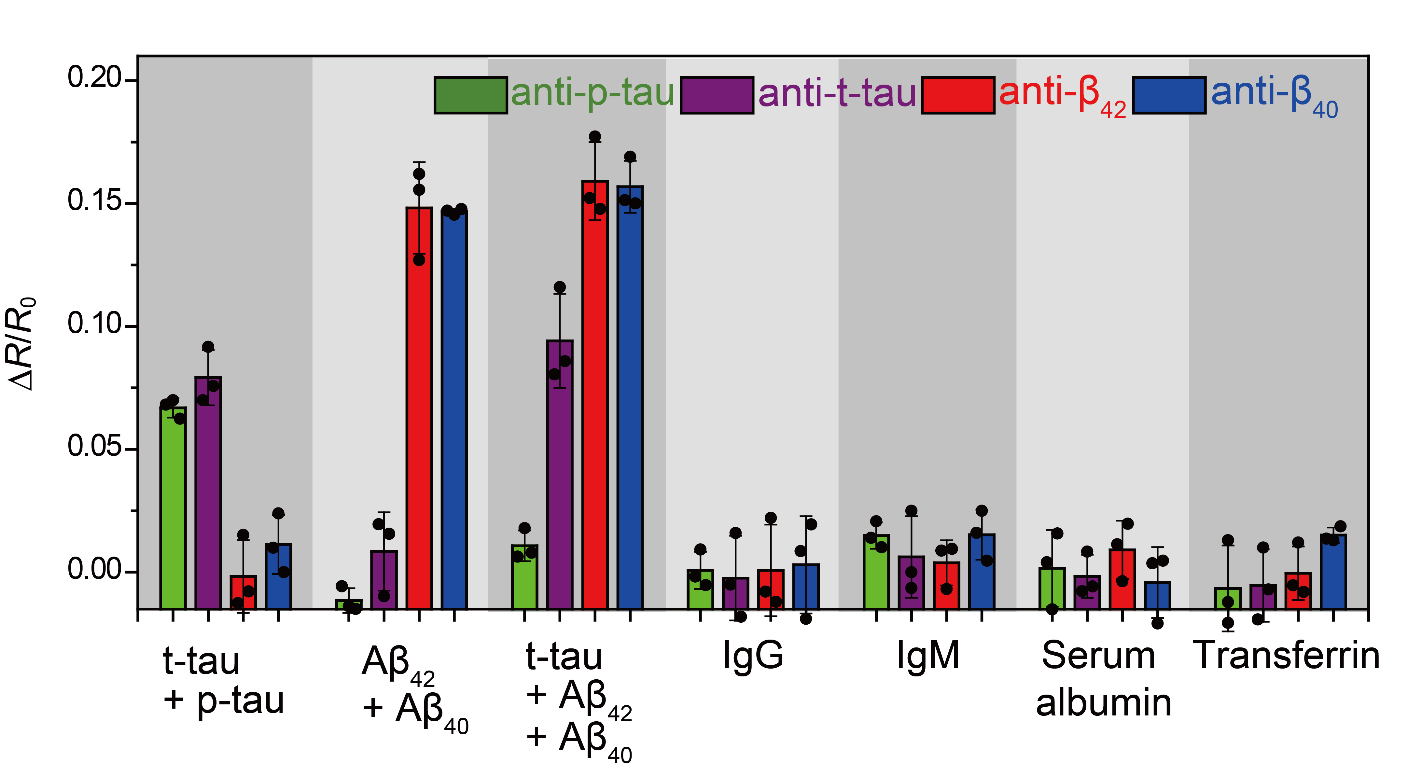
**

**Supplementary Figure 26**. Changes in the resistance of the densely aligned CNT sensor array upon exposure to various individual AD biomarkers and their mixtures. The concentrations of Aβ_42_ and Aβ_40_ were 22.2 fM, 23.1 fM, respectively. In case of t-tau and p-tau, the concentrations were 21.8 fM and 360 fM, respectively. The concentrations of IgG, IgM, serum albumin, and transferrin were 1 nM. The measurement was performed in triplicate and all the values represent the mean ± standard deviation. Source data are provided as a Source Data file.

**
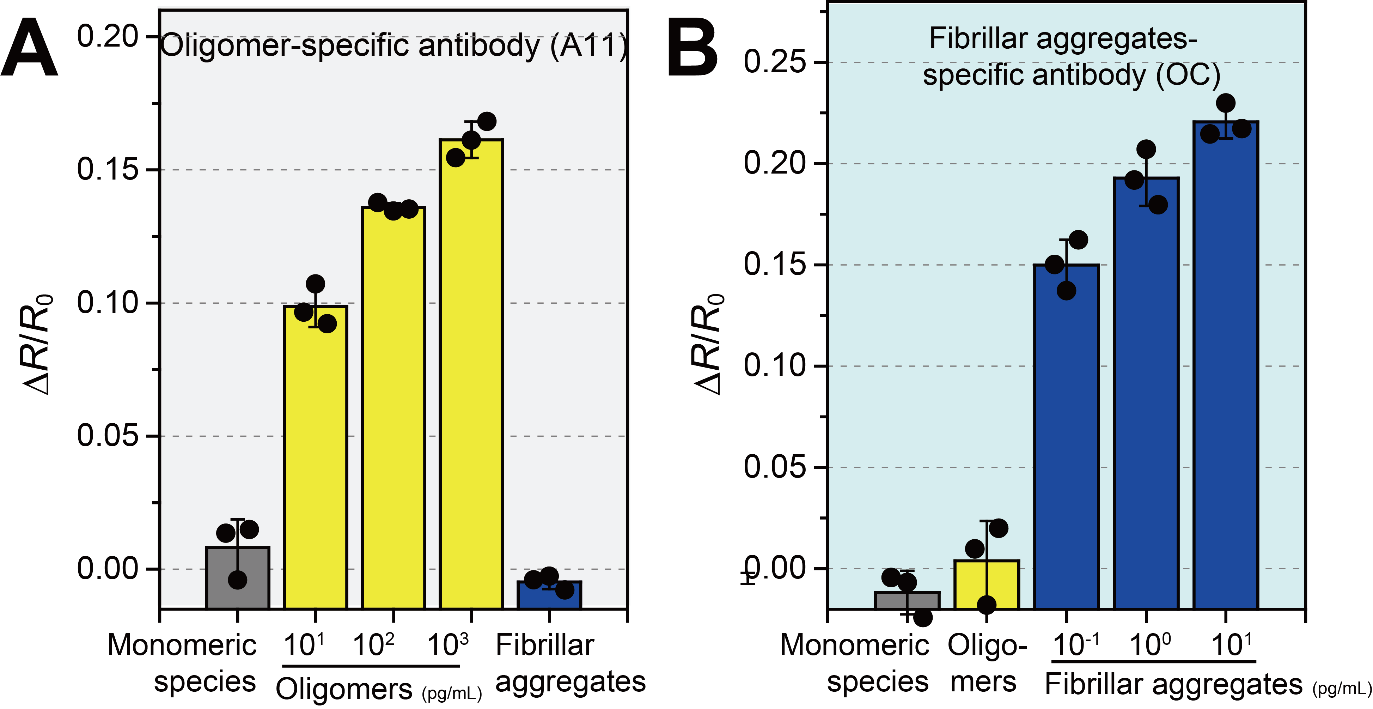
**

**Supplementary Figure 27**. Changes in resistance of the densely aligned CNT sensor array upon exposure to different Aβ aggregate species. We prepared oligomeric and fibrillar Aβ species according to the literature (*Science* 2003, **300**, 486; *J. Biol. Chem.* 2007, **282**, 1031) and modified the CNT channels using (A) A11 antibody and (B) OC antibody. A11 antibody recognizes generic epitopes of Aβ oligomers, but does not bind to monomeric or fibrillar species. In contrast, OC antibody binds to Aβ fibrillar aggregates only. The measurements were performed in triplicate and all the values represent the mean ± standard deviation. Source data are provided as a Source Data file.


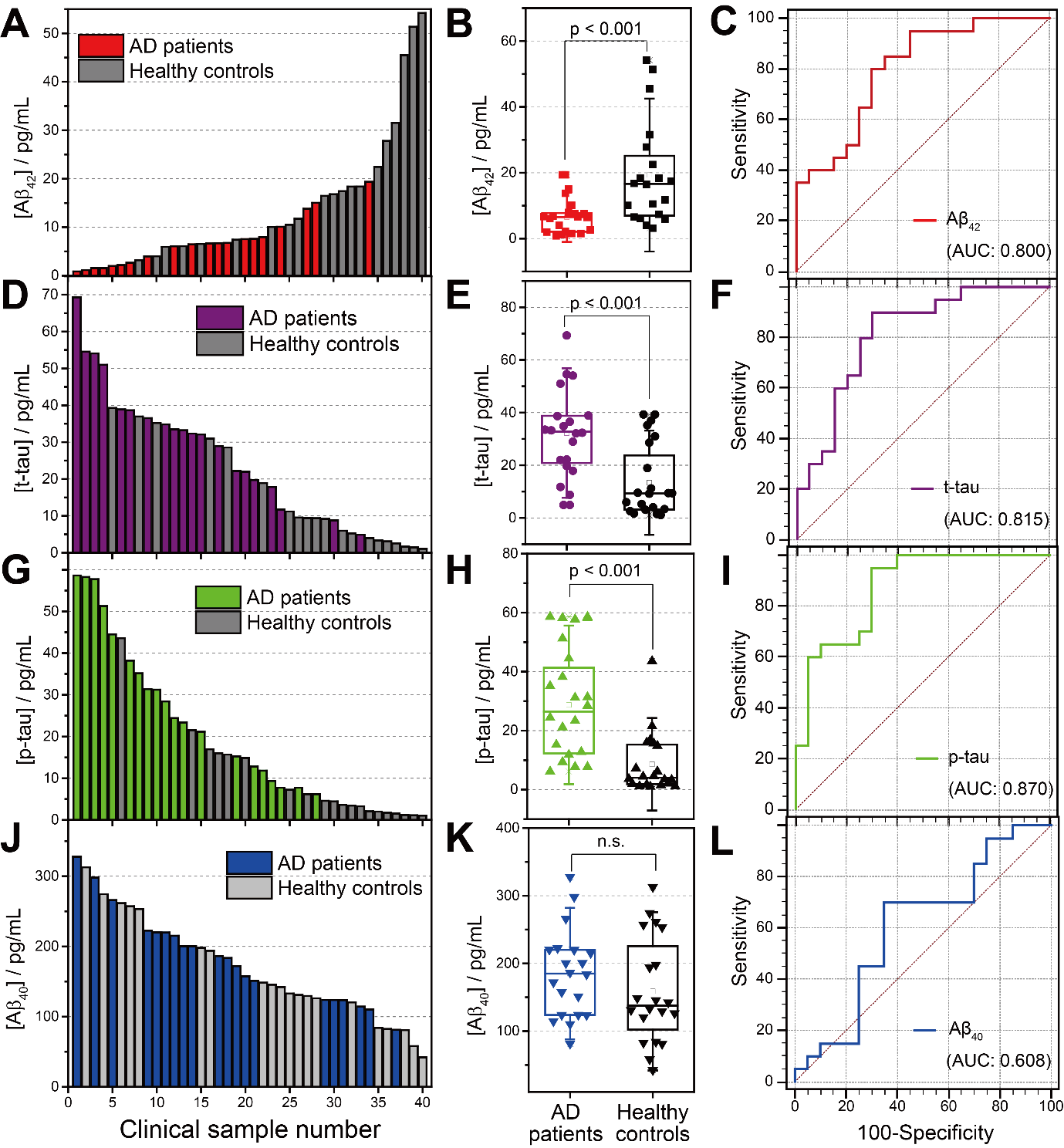


**Supplementary Figure 28.** Waterfall plots, box plots, and ROC curves showing the measured levels and AUC values of (A,B, and C) Aβ_42_, (D,E, and F) t-tau, (G, H, and I) p-tau, and (J, K, and L) Aβ_40_ in the plasma of AD patients (n = 20) and healthy controls (n = 20). In the boxes, the 25th, 50th (median) and 75th percentiles of the data are indicated. The whiskers represent mean ± 1.5 standard deviation. Significant differences are indicated by p-value. Statistical analysis was carried out by means of one-way analysis of variance (ANOVA). n.s. not significant. Source data are provided as a Source Data file.

***
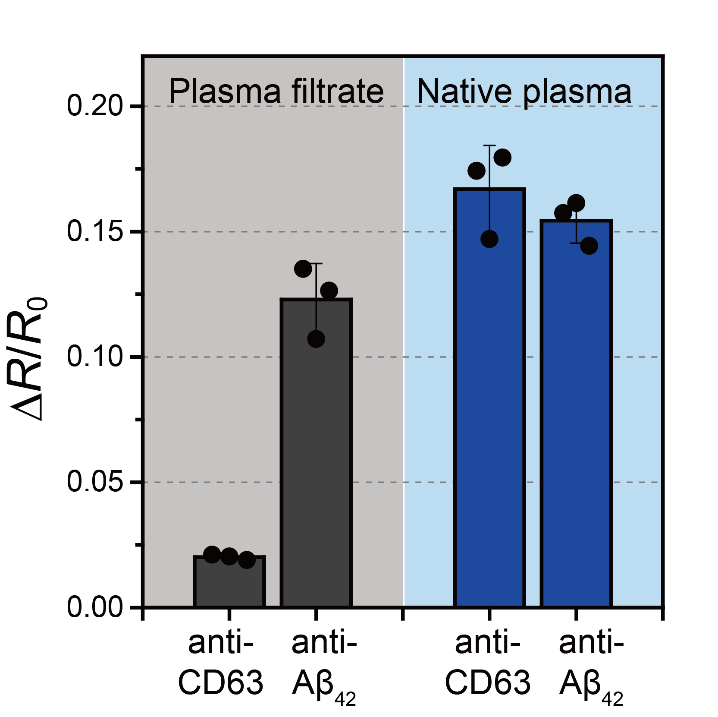
***

**Supplementary Figure 29**. Changes in the resistance of sensor array upon exposure to plasma filtrate and native clincial plasma samples. Our sensor array can detect both unbound Aβ_42_ and Aβ_42_ bound with other proteins in blood plasma. The antibody targeting CD63 proteins was obtained from BD Biosciences, USA (Cat#. 556019). We used the 12F4 Antibody to target Aβ_42_. The measurements were performed in triplicate and all the values represent the mean ± standard deviation. Source data are provided as a Source Data file.

**
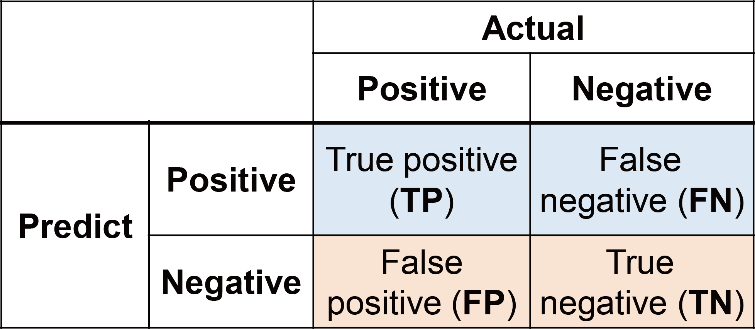
**

**Supplementary Figure 30.** Diagnostic parameters used for plotting ROC curves. The sensitivity, selectivity, and accuracy were computed as follows: sensitivity=TP/(TP+FN), selectivity= TN/(TN+FP), accuracy=(TP+TN)/(TP+TN+FP+TN). Each point on a ROC curve represents a true positive rate (i.e., sensitivity) and the false positive rate (i.e., 1-specificity) obtained at a specific threshold concentration. The closer the ROC curve is located to the upper left corner, the higher the diagnostic accuracy of the sensing platform becomes.**
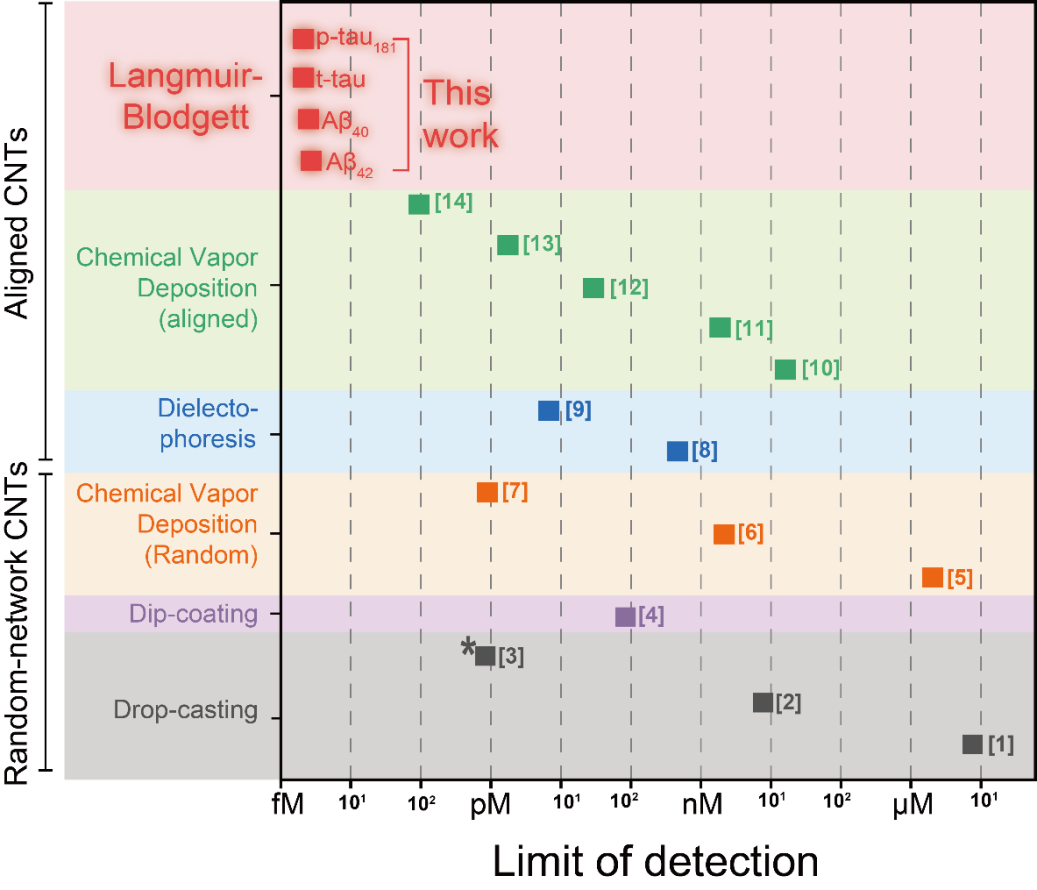
**

**Supplementary Figure 31.** Limit of detections of CNT-based biosensors reported so far. We classified the references according to the fabrication technique of CNT-based transducers. The molar concentrations indicated in papers were converted into the molarity using molecular weight of each biomarker. We indicated the lowest LOD value for the studies where multiple values of LOD were reported. References indicated in the plots are listed at the end of the Supplementary Information file. * In the case of [3], the value of LOD is marked based on the measured values without additional amplifiers. According to this reference, the sensing signal does not appear at the IgG concentration of 80 pg/mL or less.

**
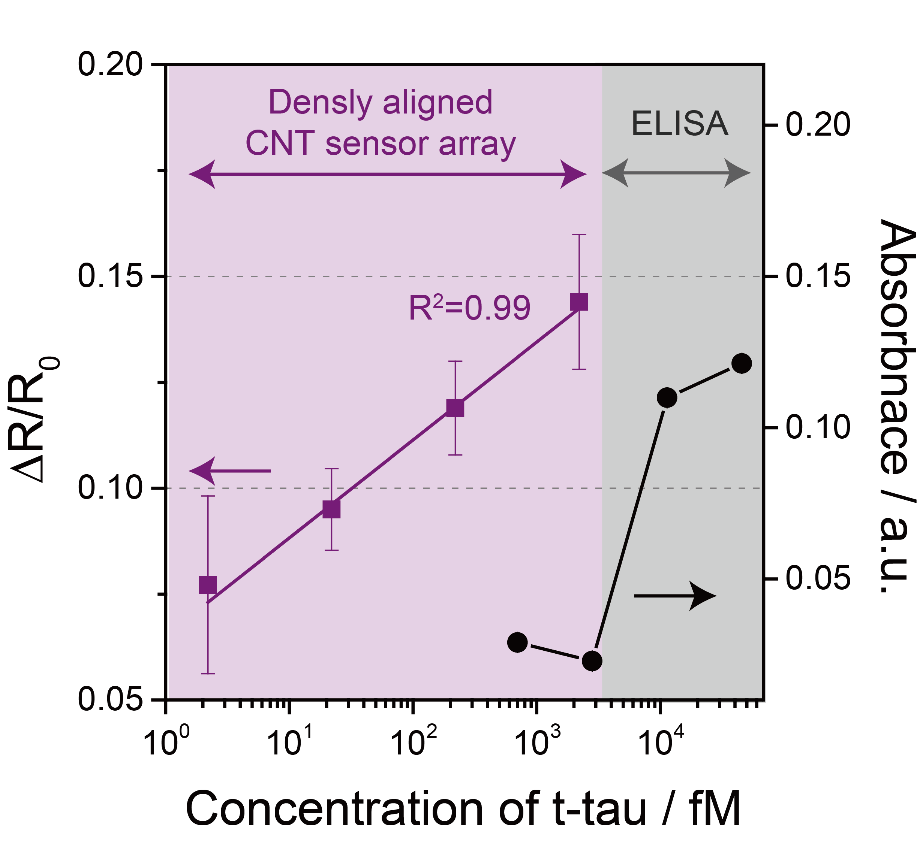
**

**Supplementary Figure 32**. Comparison of sensing signals between densely aligned CNT sensor array and ELISA. The ELISA was less sensitive in detecting t-tau proteins in plasma than the densely aligned CNT sensor array; the measurable concentrations of ELISA were above 2.72 pM whereas linear dependences of the array’s resistance change on the t-tau proteins’ logarithmic concentrations (R^2^ > 0.99) within the concentrations ranging from 2.1 fM to 2.1pM. The measurements were performed in triplicate and all the values represent the mean ± standard deviation. Source data are provided as a Source Data file.

**
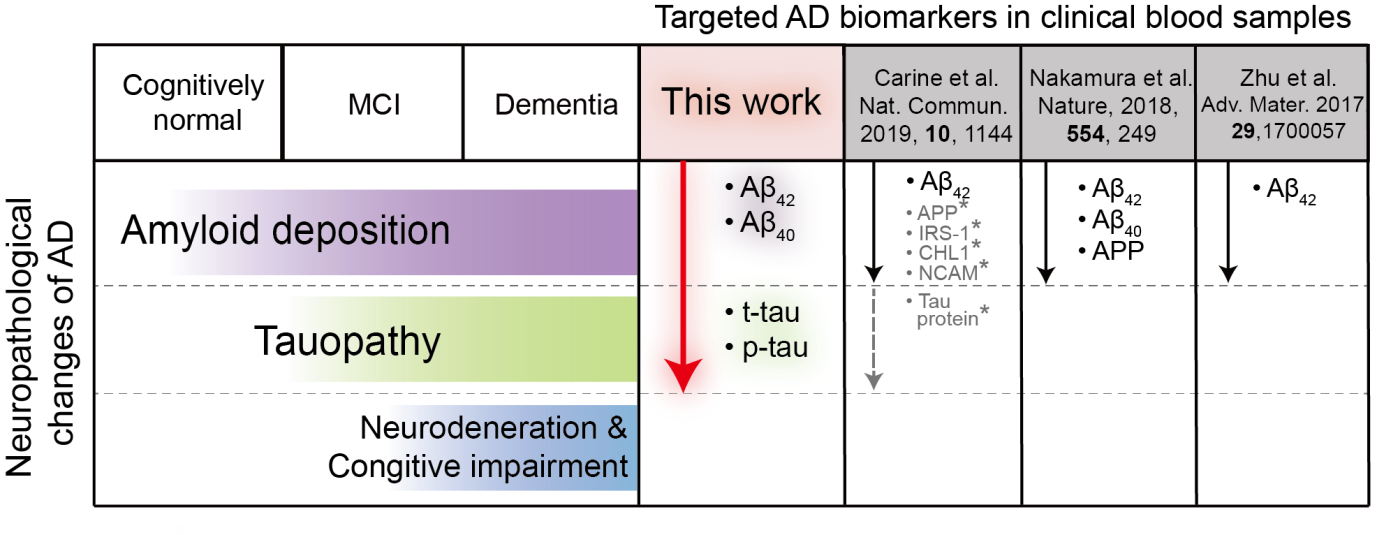
Supplementary Figure 33**. List of AD biomarkers detected in clinical blood using our densely aligned CNT device and previously reported sensing platforms (*Nat. Commun*. 2019, 10, 1144; *Nature*, 2018, 554, 249; *Adv. Mater.* 2017, 29,1700057). Our densely aligned CNT sensor array can accurately detect tau proteins (t-tau and p-tau) as well as Aβ peptides in clinical blood samples. APP: amyloid precursor protein, IRS-1: insulin receptor substrate 1, CHL1: close homolog of L1, NCAM: neuronal cell adhesion molecule. *In the report, only Aβ_42_ was analyzed in the clinical samples, though the sensing platform’s sensitivities toward APP, IRS-1, CHL1, NCAM and tau proteins were verified in defined solutions.

**
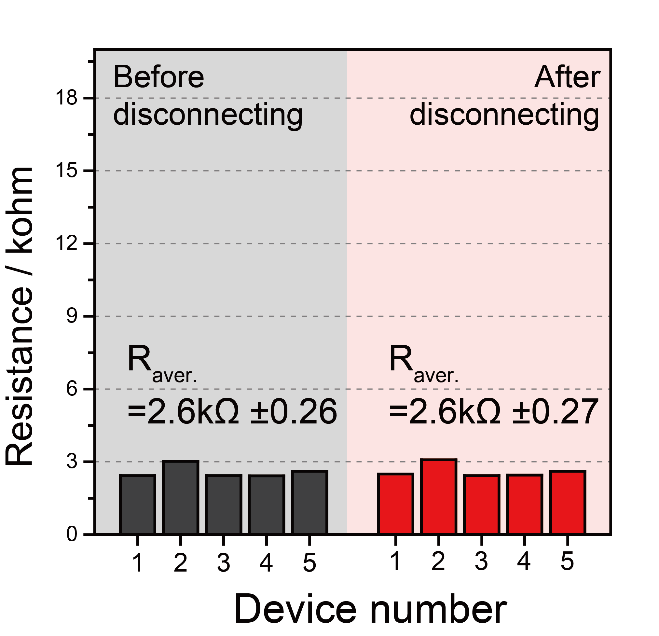
**

**Supplementary Figure 34.** Resistance of each CNT device in sensor chip before and after disconnecting adjacent devices. The resistances of the CNT devices were similar before and after disconnecting the films. Source data are provided as a Source Data file.


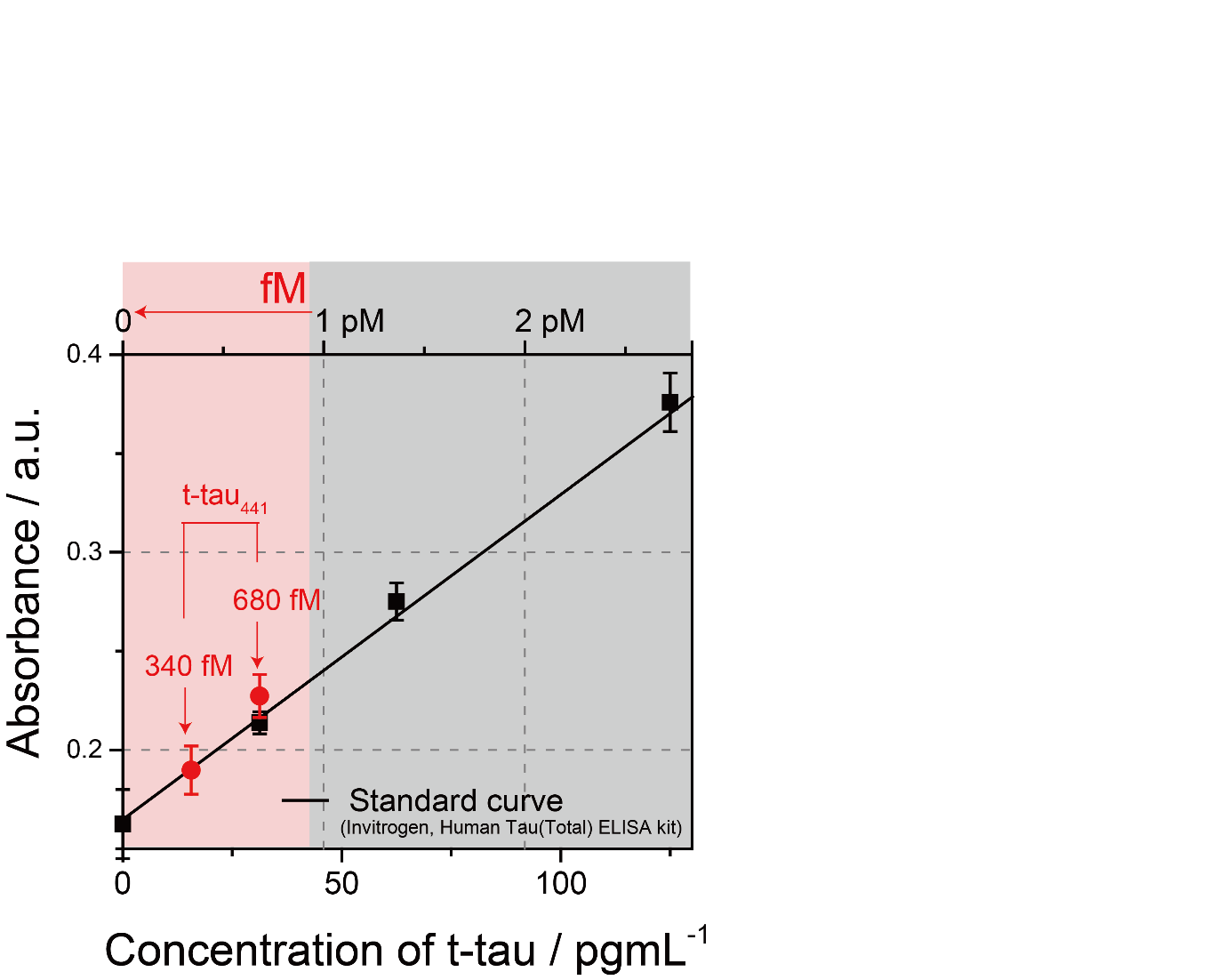


**Supplementary Figure 35**. Quantification of t-tau concentration using a standard curve of ELISA. The estimated concentrations of t-tau protein samples (nominal concentrations: 340, 680 fM) were 310.675 fM ± 106 and 718.9 fM ±170, respectively. The measurements were performed in triplicate and all the values represent the mean ± standard deviation. Source data are provided as a Source Data file.

**Supplementary Table 1**. List of target biomarkers and the corresponding bio-receptors employed in the densely aligned CNT sensor array.

| **Target** | **Antibody**  **(Manufacturer, catalog no.)** | **Description** |
| --- | --- | --- |
| Aβ_42_ | Clone 12F4  (Biolegend, Cat #. 805501) | Reactive to the C-terminus of β-amyloid and is specific for the isoform ending at the 42nd amino acid. |
| Aβ_40_ | Clone 11A50-B10 (Biolegend, Cat #. 805401) | Reactive to the C-terminus of β-amyloid and is specific for the isoform ending at the 40th amino acid. |
| t-tau | Clone Tau-5  (Millipore, Cat #. 577801) | Recognizes the phosphorylated and unphosphorylated isoforms of tau proteins. The epitope of Tau-5 lies within amino acids 210-241of tau proteins. |
| p-tau_181_ | Clone M7004D06  (Biolegend, Cat #. 80846602) | Recognizes the human tau phosphorylated at 181 residue |

**Supplementary Table 2.** Summary of sensing performance of the densely aligned CNT sensor array toward AD biomarkers.

| **Validation parameter** | | **AD biomarkers** | | | |
| --- | --- | --- | --- | --- | --- |
|  |  | **Aβ_42_** | **Aβ_40_** | **t-tau** | **p-tau** |
| Precision | Coefficient of variation, % | 5.24  (1.46-8.60) | 5.16  (1.72-8.73) | 6.92  (2.53-8.78) | 6.36  (3.39-8.58) |
| Sensitivity | Limit of detection, fM | 2.13 | 2.20 | 2.45 | 2.72 |
|  | Limit of quantification, fM | 9.86 | 10.9 | 15.1 | 20.7 |
| Accuracy | Spike recovery*,%  (range) | 93.0  (87.3-99.5) | 97.4  (91.0-103) | 97.6  (91.7-102.1) | 96.4  (91.1-98.8) |

*in human plasma

**Supplementary Table 3.** Demographic characteristics of the subjects.

|  | **AD patients** | **Healthy controls** |
| --- | --- | --- |
| Numbers | 20 | 20 |
| Male/Female | 9/11 | 11/9 |
| Ages mean ± SD  (range) | 72.5 ± 4.38  (65-81) | 70.8 ± 3.07  (67-79) |

**Supplementary Table 4.** Summary of the average concentrations of AD biomarkers measured in clinical plasma samples using a densely aligned CNT sensor array.

|  | **AD biomarkers** | | | |
| --- | --- | --- | --- | --- |
|  | **Aβ_42_** | **Aβ_40_** | **t-tau** | **p-tau_181_** |
| AD patients, pgmL^-1^ | 6.49 ± 5.02 | 184 ± 67.8 | 32.2 ± 16.4 | 28.7 ± 17.9 |
| Healthy controls, pgmL^-1^ | 19.3 ± 15.5 | 159 ± 78.0 | 13.4 ± 13.2 | 8.60 ± 10.5 |

**Supplementary Table 5.** Summary of diagnostic performances of the multiplexed, densely aligned CNT sensor array.

| **Biomarkers** | **Cut-off**  **value** | **Sensitivity*, %** | **Selectivity*, %** | **Accuracy*, %** | **AUC** |
| --- | --- | --- | --- | --- | --- |
| Aβ_42_ | ≤7.94 | 80.0 | 70.0 | 73.2 | 0.800 |
| t-tau | >11.2 | 90.0 | 70.0 | 80.5 | 0.815 |
| p-tau | >7.23 | 95.0 | 70.0 | 80.5 | 0.870 |
| Aβ_42_/ Aβ_40_ | ≤0.075 | 90.0 | 90.0 | 87.8 | 0.925 |
| t-tau/Aβ_42_ | >2.34 | 90.0 | 90.0 | 90.2 | 0.955 |
| p-tau/ Aβ_42_ | >2.14 | 90.0 | 90.0 | 87.8 | 0.942 |

*Sensitivity = ratio of true positives to total positive values, Selectivity = the ratio of true negatives to total negative values. Accuracy= ratio of total true values to total values. The cut-off values were determined by Youden index

**Supplementary Table 6.** Comparison between the electrical detection method and other analytic techniques.

| **Category** | **Electrical detection**  **(This work)** | | **Optical detection**  (*Nat. Commun.* 2019, **10**, 1144; *Adv. Mater.* 2017, **29**,1700057) | | **Mass spectroscopy**  (*Nature*, 2018, **554**, 249) |
| --- | --- | --- | --- | --- | --- |
| Description | The target biomarkers are detected by measuring a change in the resistance of the transducer. | | Detection of biomarkers is accomplished by measuring the changes in the reflected light obtained on a detector | | Analytic technique that measures the mass-to charge ratio of ionized target biomarkers. |
| Equipment for measurement | Commercial multimeter | | Expensive peripheral optical equipment | | Expensive and specialized equipment |
| Usability | - High portability   (Ease to miniaturization)   - Convenience in sensing (Label-free detection) - Quick detection | - Convenience in sensing (Label-free detection*) - Quick detection | | - Takes several hours in analysis (2h ~) | |
| Sample pretreatment | None | Need to steps for exosome separation** | | Considerable sample preparation steps | |

* Label-free detection was performed in *Adv. Mater.* 2017, **29**,1700057; ** In case of the study (*Nat. Commun.* 2019, **10**, 1144), additional exosome separation steps are required before the measurement.

**Supplementary References**

[1] *ACS Appl. Mater. Interfaces*, 2015, 7, 584; [2] *Biosens. Bioelectron.* 2016, 86, 308; [3] *J. Am. Chem. Soc*. 2004, 126, 3010; [4] *Appl. Phys. Lett.* 2016, 109, 243504; [5] *Biosens. Bioelectron.* 2015, 63, 325; [6] *Proc. Natl. Acad. Sci. U. S. A*. 2003, 100, 4854; [7] *J. Am. Chem. Soc*., 2006, 128, 2188; [8] *Nano Lett*., 2018, 18, 4130; [9] *Lab Chip*, 2010, 10, 2052; [10] *J. Phys. Chem. C*, 2012, 116, 19490; [11] *Biosens. Bioelectron.* 2013, 43, 143; [12] *Sens. Actuators, B*, 2017, 249, 691; [13] *Biosens. Bioelectron.* 2010, 25, 1989; [14] *ACS Appl. Mater. Interfaces*, 2016, 8, 9600.
